# Phenotyping of UGT1A1 Activity Using Raltegravir Predicts Pharmacokinetics and Toxicity of Irinotecan in FOLFIRI

**DOI:** 10.1371/journal.pone.0147681

**Published:** 2016-01-25

**Authors:** Lawrence Soon-U Lee, Kok-Yong Seng, Ling-Zhi Wang, Wei-Peng Yong, Kim-Hor Hee, Thomas I. Soh, Andrea Wong, Pei F. Cheong, Richie Soong, Nur S. Sapari, Ross Soo, Lu Fan, Soo-Chin Lee, Boon C. Goh

**Affiliations:** 1 Department of Medicine, National University Health System, Singapore, Singapore; 2 Department of Medicine, Yong Loo Lin School of Medicine, National University of Singapore, Singapore, Singapore; 3 Department of Pharmacology, National University of Singapore, Singapore, Singapore; 4 Cancer Science Institute, National University of Singapore, Singapore, Singapore; 5 Department of Haematology-Oncology, National University Health System, Singapore, Singapore; Imperial College London, UNITED KINGDOM

## Abstract

**Background:**

Irinotecan toxicity correlates with UGT1A1 activity. We explored whether phenotyping UGT1A1 using a probe approach works better than current genotyping methods.

**Methods:**

Twenty-four Asian cancer patients received irinotecan as part of the FOLFIRI regimen. Subjects took raltegravir 400 mg orally and intravenous midazolam 1 mg. Pharmacokinetic analyses were performed using WinNonLin and NONMEM. Genomic DNA was isolated and screened for the known genetic variants in UGT1A1 and CYP3A4/5.

**Results:**

SN-38G/SN-38 AUC ratio correlated well with Raltegravir glucuronide/ Raltegravir AUC ratio (r = 0.784 p<0.01). Midazolam clearance correlated well with irinotecan clearance (r = 0.563 p<0.01). SN-38 AUC correlated well with Log10Nadir Absolute Neutrophil Count (ANC) (r = -0.397 p<0.05). Significant correlation was found between nadir ANC and formation rate constant of raltegravir glucuronide (r = 0.598, P<0.005), but not UGT1A1 genotype.

**Conclusion:**

Raltegravir glucuronide formation is a good predictor of nadir ANC, and can predict neutropenia in East Asian patients. Prospective studies with dose adjustments should be done to develop raltegravir as a probe to optimize irinotecan therapy.

**Trial Registration:**

Clinicaltrials.gov NCT00808184

## Introduction

The topoisomerase-I inhibitor irinotecan was approved in the United States for the second-line treatment of patients with metastatic colorectal carcinoma. Currently, irinotecan is approved as a single agent and in combination therapy with other drugs such as fluorouracil, oxaliplatin, and bevacizumab in different first-line and second-line regimens for the treatment of gastrointestinal malignancies [1].

Several metabolic enzymes are involved in the elimination of irinotecan and its active metabolite SN-38. Irinotecan is cleared by members of the cytochrome P450 3A (CYP3A) enzyme and converted to SN-38 via human carboxyl esterase (hCE) [2] while SN-38 is cleared by the uridine-diphosphate glucuronosyltransferase 1A (UGT1A) family [3]. Because the functions of these enzymes are affected by environmental and genetic factors, the pharmacokinetics of irinotecan and its metabolites vary greatly between patients.

Like most cytotoxic agents, irinotecan has a narrow therapeutic window and causes treatment limiting toxicities such as neutropenia and diarrhea [4]. Therefore, the large interindividual variability may result in unacceptable side effects in some patients and in diminished therapeutic effects in others. New dosing strategies that take the pharmacologic profile of irinotecan in the individual patient into account could potentially replace conventional body surface area or flat fixed dosing, if this would lead to a reduction in the pharmacokinetic variability.

Thus far, dosing strategies have mainly focused on polymorphisms affecting the expression of enzymes involved in the metabolism of SN-38, such as *UGT1A1* polymorphisms [5,6]. The UGT1A1*28 polymorphism involves a 7 TA repeats compared to the wild type of 6 repeats in the promoter region and reduces the expression of UGT1A1 enzyme; it has been associated with slower SN-38 glucuronidation and greater neutrophil toxicity following irinotecan exposure [7]. Therefore, genotyping of UGT1A1 is recommended before treatment with irinotecan, with dose reduction for patients homozygous for UGT1A1*28 [8]. However, the expression of these enzymes is also influenced by environmental factors, implying that dose-individualization strategies should not solely focus on inherited variables. Furthermore, UGT1A1*28 has a higher allelic frequency in Western populations compared to East Asians [9].

A new dosing algorithm was created and validated involving phenotyping with midazolam as a CYP3A probe. This approach was found to reduce the interindividual variability by 19%, but this reduction was not statistically significant [10]. This lack of significance could be due to the fact that CYP3A only clears the parent irinotecan compound and not the active metabolite, and therefore the correlation is less direct.

We developed a new phenotyping method which aims to individualize irinotecan therapy based on UGT1A1 instead of CYP3A. Raltegravir is an antiretroviral drug which is predominantly metabolized by UGT1A1 to its glucuronide, and we hypothesize that clearance of raltegravir would correlate better with irinotecan toxicity since SN-38, the active metabolite, is cleared by UGT1A1. We also hypothesized that phenotyping by UGT1A1 would correlate better with toxicity than genotyping, particularly in East Asians where genotyping strategies are lacking given the low frequency of UGT1A1*28, and the lower influence of a more common polymorphism, UGT1A1*6, on irinotecan induced toxicity. The population of patients we studied were colorectal cancer patients receiving FOLFIRI, a very commonly used chemotherapeutic regimen containing irinotecan and 5-Fluorouracil.

## Materials and Methods

### Patients

**Fig 1:**
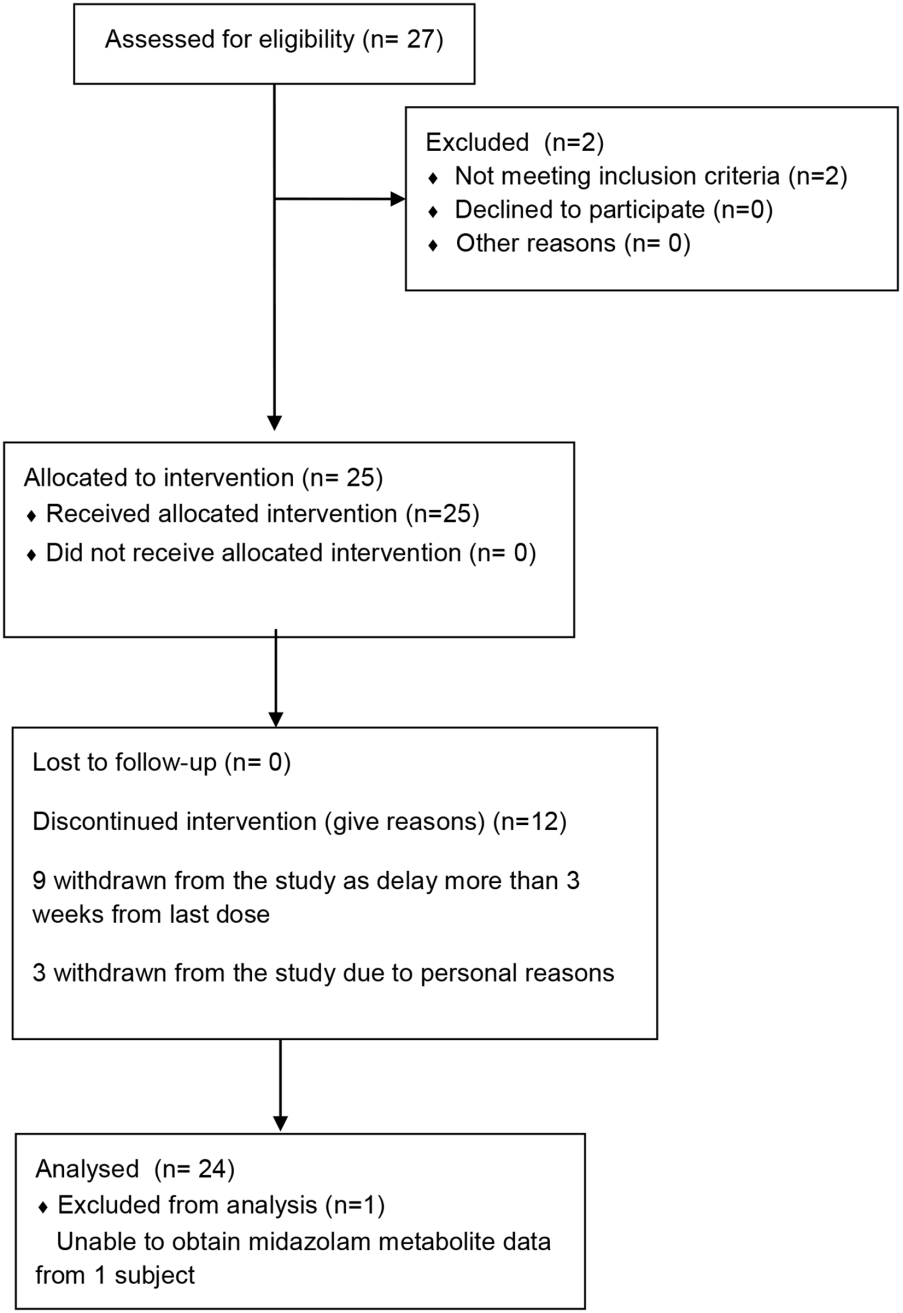
CONSORT Flow Diagram for trial.

Twenty-four Asian patients with advanced stage cancer requiring systemic FOLFIRI chemotherapy (folinic acid, 5-fluorouracil and irinotecan) were recruited for this study between 29 September 2009 and 4 July 2011 (Fig 1). Inclusion criteria included: (a) Histologically or cytologically proven solid tumour for which irinotecan given by the FOLFIRI regimen is indicated and prescribed by the attending physician, (b) Age above 21 years, (c) Karnofsky performance status > 70%. (d) WBC > 3.0 x 10^3^ /μL; ANC > 1500 x 10^3^ /μL, Hemoglobin > 9.0 g/dl and Platelets > 100000/μl, (d) Creatinine < 1.5 x ULN or calculated creatinine clearance > 40 ml/min, (e) Total bilirubin < 1.5 x ULN, SGOT, SGPT > 2.5 x ULN, or > 5 x ULN with liver metastases. Exclusion criteria included: (a) Biologic therapy or chemotherapy within 4 weeks, (b) Radiation therapy within 4 weeks if > 25% of bone marrow was irradiated. (c) Have received any medications that are known to be metabolised by UGT1A1 within 30 days of the first dose of irinotecan. (d) Short gut syndrome or other causes of malabsorption. (e) Colony stimulating factors within 2 weeks. (f) Women of childbearing potential not practicing birth control and pregnant women, and (g) Rapidly progressive intracranial or spinal metastatic disease. All participants gave informed written consent prior to any study procedures. The study was reviewed and approved by the Institutional Review Board (IRB) of the National Healthcare Group, Singapore. This trial was registered at ClinicalTrials.gov (NCT00808184).

### Treatment

Subjects were administered raltegravir 400 mg orally (as a UGT1A1 probe) and intravenous midazolam 1 mg (as a CYP3A4 probe) one day before the first dose of their chemotherapy. In the fasted state. Serial blood samples were taken from the patients at baseline, and at 0.5, 1, 2, 4, 6, 8 and 24 hours after administration of the probe drugs.

The next day, FOLFIRI were administered as irinotecan 180 mg/m^2^ in 250 mL Normal Saline over 90 min followed by Leucovorin at 400 mg/m^2^ in 250 mL Normal Saline over 2 hours followed by 5-Flourouracil 400 mg/m^2^ IV bolus followed by 5-Flourouracil 2400 mg/m^2^ over 46 hours. Premedications were administered as per routine clinical practice. Cycles were repeated every 2 weeks, and dose delay for 1 week was instituted for patients who had persistent neutropenia or treatment related diarrhea.

Blood was collected at baseline, prior to the end of infusion and 0.5, 1, 2, 4, 6, 8, 22, 24, and 28 hours after the irinotecan infusion for pharmacokinetic analyses.

### Toxicity evaluation

Patients were seen weekly at the outpatient clinic for follow-up, which included a physical examination and routine hematologic, renal, and hepatic laboratory analyses. All side effects, including leukopenia, neutropenia, and late-onset diarrhea were graded using the National Cancer Institute Common Terminology Criteria for Adverse Events version 3. The nadir absolute neutrophil count was also calculated from each patient to monitor neutropenia objectively.

### Pharmacokinetic Analyses

The concentrations of midazolam and 1-hydroxy-midazolam in the patient plasma were measured by a LC-MS/MS method validated in our laboratory [11]. Briefly, 100 μL of plasma samples and 300 μL of methanol containing the internal standards were extracted using Captiva^™^ ND^Lipids^, a non-drip 96-well solid phase extraction. Filtrates were collected in the 96-well collection plate and transferred to the autosampler of the UHPLC. Five microliter of the filtrate was injected into LC-MS/MS system for analysis. For the preparation of standard curves, standard stock solutions were prepared in methanol as 1 mg/mL and stored at –20°C. Highest calibration standards were prepared by spiking the respective standard stock solutions in blank plasma. This was followed by serial dilution with blank plasma of the highest calibration standard to obtain various calibration ranges for each analyte.

The LC-MS/MS system consisted of an Agilent 1290 binary pump equipped with a cooled autosampler (6°C) connected to Agilent 6460 triple quadrupole mass spectrometer (Agilent Technologies, Wald-bronn, Germany). Chromatographic separations were achieved on a ZORBAX Eclipse Plus C18 Rapid Resolution HD (Agilent, 50 mm × 2.1 mm, 1.8 μm) with gradient elution. Mobile phases A and B were water and 90% (v/v) acetonitrile (HPLC grade, Merck), respectively, both containing 0.1% formic acid (AR, Sigma). The mass spectrometer was operated under positive ionization mode and the detection was based on the multiple-reaction monitoring of m/z. The method has been validated according to FDA guidance for accuracy (90.2–110.5%) and precision (CV < 8.7%). Stability of the analytes has also been assessed (mean recovered concentration of 93.7–111.7%) after 48 h storage at 6°C autosampler and two freeze-thaw cycles.

The concentrations of irinotecan, SN-38 and SN-38 glucuronide were measured by a HPLC method, modified from the method previously described in [12]. The precision was 4.2–7.0% for irinotecan and 4.4–8.6 for SN-38, respectively. The accuracy was 99.2–104.6% for irinotecan and 100.1–103.8% for SN-38.

The concentrations of raltegravir and raltegravir glucuronide in the patient plasma were measured by a LC-MS/MS method previously described in [13]. This method was able to not only quantify raltegravir but also the main metabolite, the glucuronide, allowing the UGT1A1 pathway to be elucidated. The precision was 1.6–6.6% for raltegravir and 2.1–6.9 for raltegravir glucuronide, respectively. The accuracy was 98.6–106.1% for raltegravir and 96.3–100.3% for raltegravir glucuronide.

Non-compartmental analyses were performed using Phoenix WinNonLin version 6 (Certara, NC, USA). For each patient and analyte, area under the curve (AUC) and clearance (CL) were derived.

### Identification of Genotypes

DNA was extracted from whole blood using the Omega Biotek E.Z.N.A Blood DNA kit (Norcross, GA, USA) according to manufacturer’s protocol. Genotyping was carried out using the Sequenom MassARRAY platform and the iPLEX ADME PGx panel according to the manufacturer’s protocols (Sequenom, San Diego, CA). The iPLEX ADME PGx panel consists of 200 pre-designed SNP and CNV assays in 36 pharmacogenetically relevant genes in 8 multiplex reactions. In brief, 20ng of genomic DNA were amplified in a multiplex PCR reaction and treated with shrimp alkaline phosphatase post-PCR to inactivate the unincorporated nucleotides. This was followed by a single base extension reaction using the IPLEX chemistry, treatment of the resin to remove salts contaminants and spotting onto the SpectroCHIP II. The genotypes were resolved by MALDI-TOF mass spectrometry and data was analysed by the TYPER 4.0.20 software (Sequenom). None of the patients carried variants for CYP3A4 polymorphisms found on the iPLEX panel and hence, this pharmacogenetic covariate was omitted from covariate model building.

To genotype UGT1A1*28 at the gene promoter which includes the TATA box1, PCR primer pairs 5’- GAGGTTCTGGAAGTACTTTGC-3’, 5’- CAGGTGCTAGGACAACTATTTC-3’ was designed and a fragment size of 458bp was amplified. Each PCR reaction was carried out in 25μl volume with 10X PCR buffer, 25mM magnesium chloride, 10mM dNTP, 2U hotstart Taq DNA polymerase, 2.5μM of each primer, and 5ng genomic DNA, with an initial denaturation step at 95°C for 5 minutes, followed by 37 cycles of denaturation at 95°C for 30secs, annealing at 54°C for 30secs, and extension at 72°C for 1 minute, followed by a final extension step at 72°C for 5 minutes. PCR products were visualized on 1.5% agarose gel. The PCR products were purified and sequenced on the ABI 3100 automated sequence analyser (Applied Biosystems Inc., Foster City,CA, USA) with the forward and reverse PCR primer.

### Population Pharmacokinetic Modelling

Plasma concentration versus time data were analysed using nonlinear mixed effects modelling in NONMEM (Version 7.2, NONMEM Project Group, San Francisco, CA) interfaced with PDx-Pop 5.0 (ICON Development Solutions, Ellicott City, MD). The first-order conditional estimation method with ε-η interaction was used to estimate the population parameters, IIV in these parameters and residual variability between measured and predicted concentrations. IIV was estimated with an exponential error model. The residual variability and its corresponding standard error were evaluated using additive error, power function, constant coefficient of variation, and additive plus proportional error models.

The adequacy of the tested models was evaluated using statistical and graphical methods [14]. The minimal value of the objective function (OFV, equal to minus twice the likelihood) provided by NONMEM was used as goodness of fit characteristic to discriminate between hierarchical models using the log likelihood ratio test. A p-value of 0.05, representing a decrease in OFV of 3.84 was considered statistically significant (chi-square distribution, degrees of freedom = 1). The covariance option in NONMEM was used to calculate estimate precisions, expressed as relative standard error (RSE). Assessment of IIV estimates and their corresponding standard errors (SE) was done to check for η shrinkage[15]. Xpose (Version 4.3.5; http://xpose.sourceforge.net/) implemented into R (Version 2.15.0; http://www.r-project.org/) were used for graphical and statistical model diagnostics.

The population pharmacokinetic model for midazolam (MDZ) and its metabolites, 1’-hydroxymidazolam (1OHM) and 1’-hydroxymidazolam glucuronide (HMG), have been previously reported by our group [16]. In brief, a two-compartment model for MDZ and two sequential compartments representing 1OHM and HMG best described the data. The conversion fraction of midazolam to its metabolite (F_1OHM/MDZ_) was assumed to be 0.6 [17]. Several covariates were found to be statistically significant in the final model: CYP3A5*3 and total bilirubin level influenced MDZ clearance; bodyweight influenced 1OHM clearance and volume; and creatinine clearance influenced HMG clearance. In this study, to calculate the rate constant for formation of 1OHM, k_12_, for each subject, the following formula was used:

$${\text{k}}_{12}=0.6\times \frac{\text{CL}}{\text{V}}.$$

where CL and V denote midazolam clearance and volume of the central compartment, respectively.

**Fig 2:**
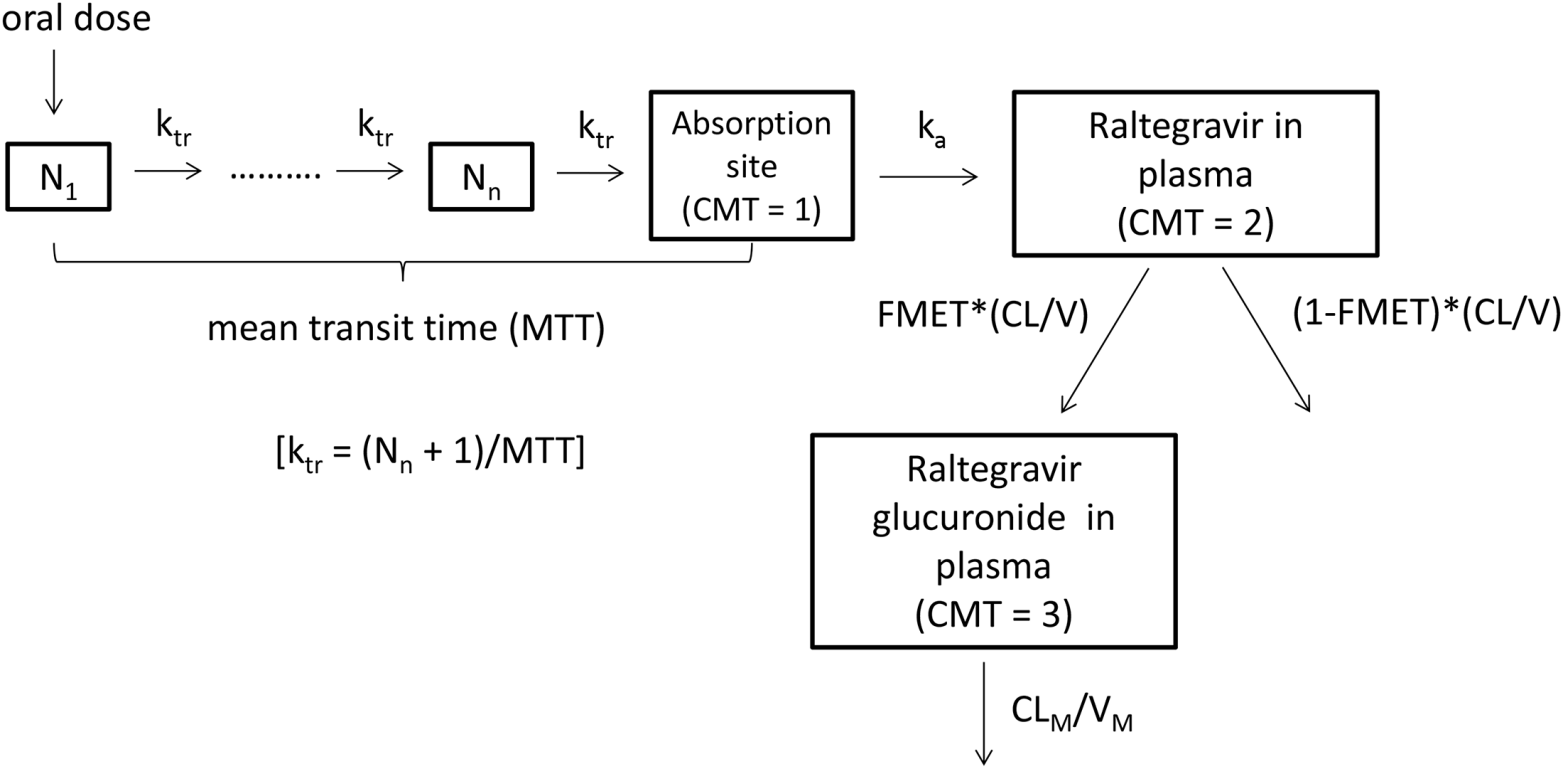
The raltegravir-raltegravir glucuronide (parent-metabolite) compartment model. N_1_ denotes the first hypothetical transit compartment up to N_n_ compartment. k_tr_ is the transit rate constant. k_a_ is the absorption rate constant from the hypothetical drug depot compartment to plasma. CL/V (or k) is the elimination rate constant of raltegravir. FMET is the fraction of raltegravir clearance for the formation of raltegravir glucuronide. V_M_, the distribution volume of the metabolite, was fixed to 1. As such, FMET is estimated as the ratio of the formation rate of glucuronide to V_M_. CL_M_ is the glucuronide clearance.

For raltegravir, the plasma pharmacokinetics were described using one depot and one central compartment, with first-order absorption and elimination based on data exploration and our previous work [18] (Fig 2). To describe the gradual and variable onset of oral raltegravir absorption, a chain of transition compartments between the depot and central compartment was tested, as previously described in [19]. The raltegravir model was first developed. Thereafter, the fixed and random effects estimates of oral clearance (CL/F), volume of distribution (V/F), first-order absorption rate constant (k_a_), mean transit time (MTT), oral bioavailability (F) and number of hypothetical transit compartments (NN) were fixed, and the glucuronide model was developed using all data. FMET, the fraction of raltegravir clearance for the formation of glucuronide, was also modelled. The distribution volume of glucuronide was fixed to 1L and FMET was interpreted as ratio of the formation rate of glucuronide to the distribution volume of the glucuronide.

Possible covariates, including, among others, patient characteristics and genotype variables (Table 1), were studied. Inter-individual variability as well as post hocs, and conditional weighted residuals (CWRES) were independently plotted against covariates to evaluate possible relationships. While categorical covariates such as sex and genotype were tested as a fraction for each category, continuous covariates were tested in a linear or power function. Covariate model building was analogous to structural model building. Potential variables were evaluated using forward inclusion and backward elimination with a level of significance of <0.05 (−3.8 points in OFV) and <0.001 (−10.8 points in OFV), respectively[18,20,21]. In addition, inclusion of a covariate in the model had to result in a decline in unexplained inter-individual variability before it was included in the final model.

**Table 1:** Patient characteristics. ALP, alkaline phosphatase; ALT, alanine transaminase; AST, aspartate transaminase; wt, wild-type genotype; het, heterozygous variant genotype; var, homozygous variant genotype.

Parameter	N = 24
*Demographics*	Median (range) or N (%)
Age (years)	59 (39–79)
Bodyweight (kg)	55 (42.4–81.1)
Height (m)	1.65 (1.47–1.79)
Body surface area (kg.m^-2^)	1.58 (1.36–2.01)
Gender: male/female	19 (79) / 5 (21)
Race: Chinese, Malay, Indian	18 (75) / 5 (21) / 1 (4)
*Clinical*	Median (range)
Serum albumin (g.L^-1^)	41.5 (25–143)
Serum creatinine (μmol.L^-1^)	74.3 (41.9–136.8)
Creatinine clearance (mL.min^-1^)	75.1 (36.7–124.3)
Total bilirubin (μmol.L^-1^)	11 (6–27)
ALP (U.L^-1^)	117.5 (57–393)
ALT (U.L^-1^)	25.5 (8–73)
AST (U.L^-1^)	32.5 (18–120)
*Genetic*	(wt/het/var) n (%)
CYP3A4*1	24 (100)
	0 (0)
	0 (0)
CYP3A5*3	6 (25)
	7 (29)
	11 (46)
UGT1A1*6	17 (71)
	7 (29)
	0 (0)
UGT1A1*28	17 (71)
	7 (29)
	0 (0)
UGT1A1*60	12 (50)
	9 (38)
	3 (12)

For each subject, using the parameter estimates from the final model, the rate constant for formation of raltegravir glucuronide, K_23_, was calculated as:

$${\text{k}}_{23}=\text{FMET}*\left(\frac{\text{CL/F}}{\text{V/F}}\right).$$

Univariate regression analyses were conducted based on the nadir absolute neutrophil count (ANC) and primary (e.g. CL) and secondary (e.g. rate constant of formation of metabolite from parent drug) pharmacokinetic parameters from the raltegravir and the midazolam population pharmacokinetic models. In addition, univariate regression analyses were also performed between the rate constants of formation of the respective metabolites and indicators of treatment toxicity, i.e. presence or absence of dose reductions or dose delays, defined as a 1 week delay in administering chemotherapy as a result of toxicity.

### Statistical considerations

Statistical calculations were done using Stata version 10 (Stata Corp, College Station, TX, USA).

To detect a moderate correlation between important parameters (r = 0.600), a sample of 19 analyzable subjects will provide 80% power to discover that the correlation is statistically different from there being no correlation at the 0.05 significance.

Pearson's correlation coefficient was used to relate two continuous variables. Differences in interindividual pharmacokinetics and nadir ANC between subjects with drug delay and subjects without drug delay were calculated. Owing to the small sample sizes in our study, all statistical analysis were conducted using either the Wilcoxon rank sum test or the Kruskal-Wallis test. Two-sided P < 0.05 values were considered significant.

## Results

### Patient characteristics

All patients received chemotherapy for metastatic gastro-intestinal malignancies. 7 patients were receiving first line chemotherapy, 13 second line, 3 third line and 1 fourth line.

Patient characteristics with descriptive statistics are presented in Table 1. For most of the patient attributes, the range was wide, for example, bodyweight was varying from 42.4 to 81.1kg, which was beneficial for the ability to identify covariate relations. One patient developed Grade 3 diarrhea and 16 developed Grade 3–4 neutropenia. 16 patients had dose delays, of whom 8 patients could not even complete 4 cycles of chemotherapy mainly due to toxicities. There were no protocol deviations.

### Non-Compartmental Analyses

SN-38G/SN-38 AUC ratio was highly correlated with Raltegravir glucuronide/ Raltegravir AUC ratio (r = 0.784 p<0.01). Midazolam clearance was highly correlated with irinotecan clearance (r = 0.563 p<0.01). SN-38 AUC correlated well with Log10Nadir Absolute Neutrophil Count (ANC) (r = -0.397 p<0.05). Neither irinotecan nor midazolam clearance correlated with Log10 Nadir ANC.

### Population pharmacokinetic analyses

The final parameter estimates for raltegravir are summarised in Table 2. Observed interindividual variability (IIV) in raltegravir plasma pharmacokinetics was moderate to high (30.5–124% CV). Accounting for the correlation between CL_RAL_/F and V_RAL_/F (correlation coefficient = 0.567) in the raltegravir model significantly improved the model. The η shrinkage values for CL_RAL_/F, V_RAL_/F, MTT, and F were 24.1, 23.7, 15.5 and 1.4% respectively. Raltegravir glucuronide pharmacokinetic data were best described by a one-compartment model with first-order clearance (Fig 2). The η shrinkage values for glucuronide CL_GLU_, and FMET were 30 and 28.3% respectively. The residual unexplained variability of raltegravir and glucuronide was best described by an additive model on log-transformed data.

**Table 2:** Final pharmacokinetic parameter estimates for the raltegravir-metabolite model.

Model parameter	Population estimate (RSE, %)
*Fixed effects*	
CL_RAL_/F (L/h)	41.7 (24.9)
MTT (h)	1.04 (25.8)
F	1 FIXED
ka (1/h)	4.23 (19.3)
NN	1.07 (15.8)
V_RAL_/F (L)	157 (32.4)
FMET	0.0324 (10.4)
CL_GLU_ (L/h)	0.715 (10.2)
V_GLU_ (L)	1 FIXED
*Random effects*	
*Interindividual variability*	
ω CL_RAL_/F (%CV)	30.5 (22.6)
ω MTT (%CV)	107.2 (41.4)
ω F (%CV)	123.7 (29.9)
ω V_RAL_/F (%CV)	81.4 (16.8)
ω FMET (%CV)	37.7 (33.2)
ω CL_GLU_ (%CV)	13.6 (47.9)
Correlation CL_RAL_/F, V_RAL_/F	0.57 (8.7)
*Residual error*	
σ RAL (SD)	0.15 (3.2)
σ GLU (SD)	0.18 (2.6)

**Fig 3:**
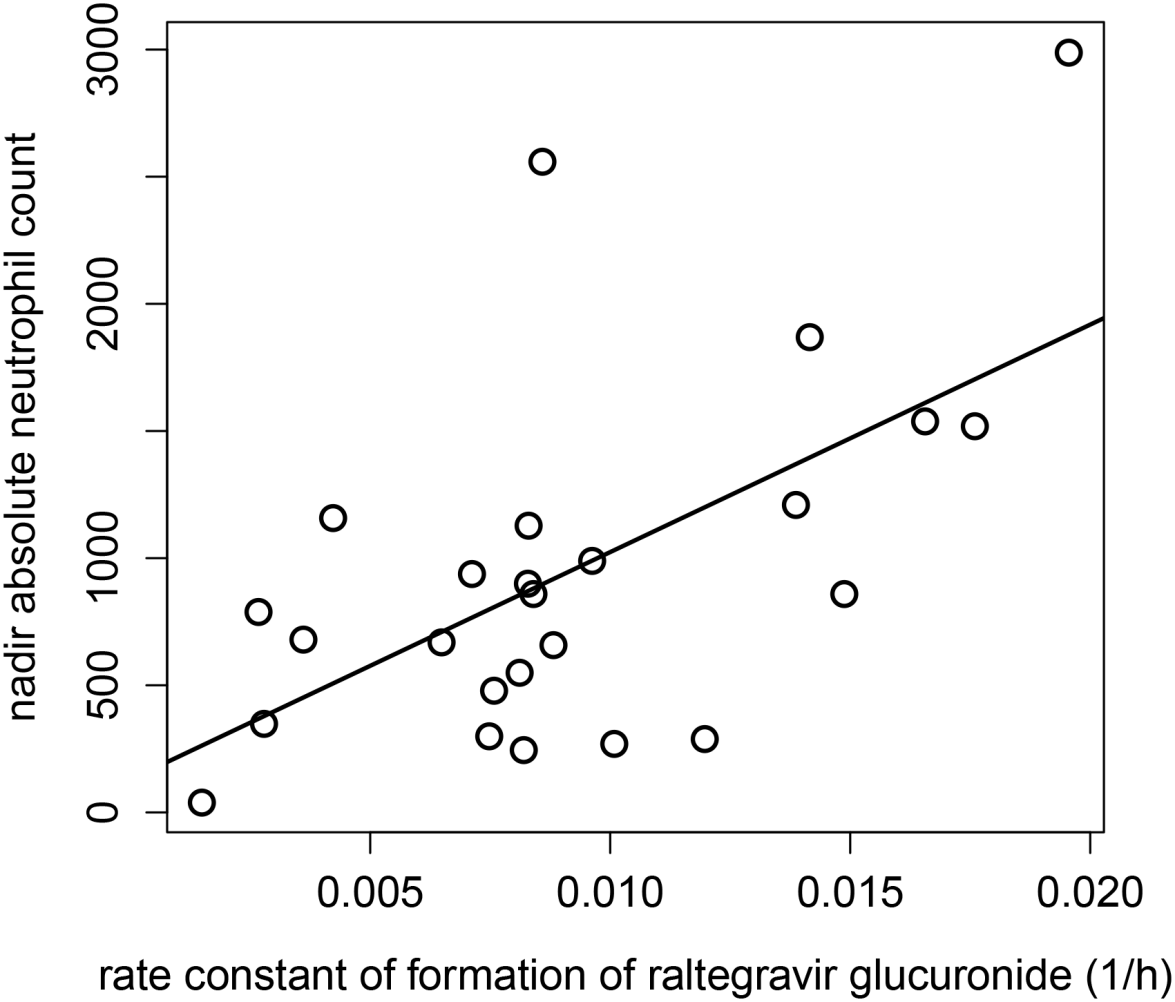
Scatterplot and linear correlation between nadir absolute neutrophil count (ANC) and rate constant of formation of raltegravir glucuronide from raltegravir (K_23_). The regression equation is ANC = 129.2 + 89461.5*K23. R is 0.598 (p = 0.00158).

**Fig 4:**
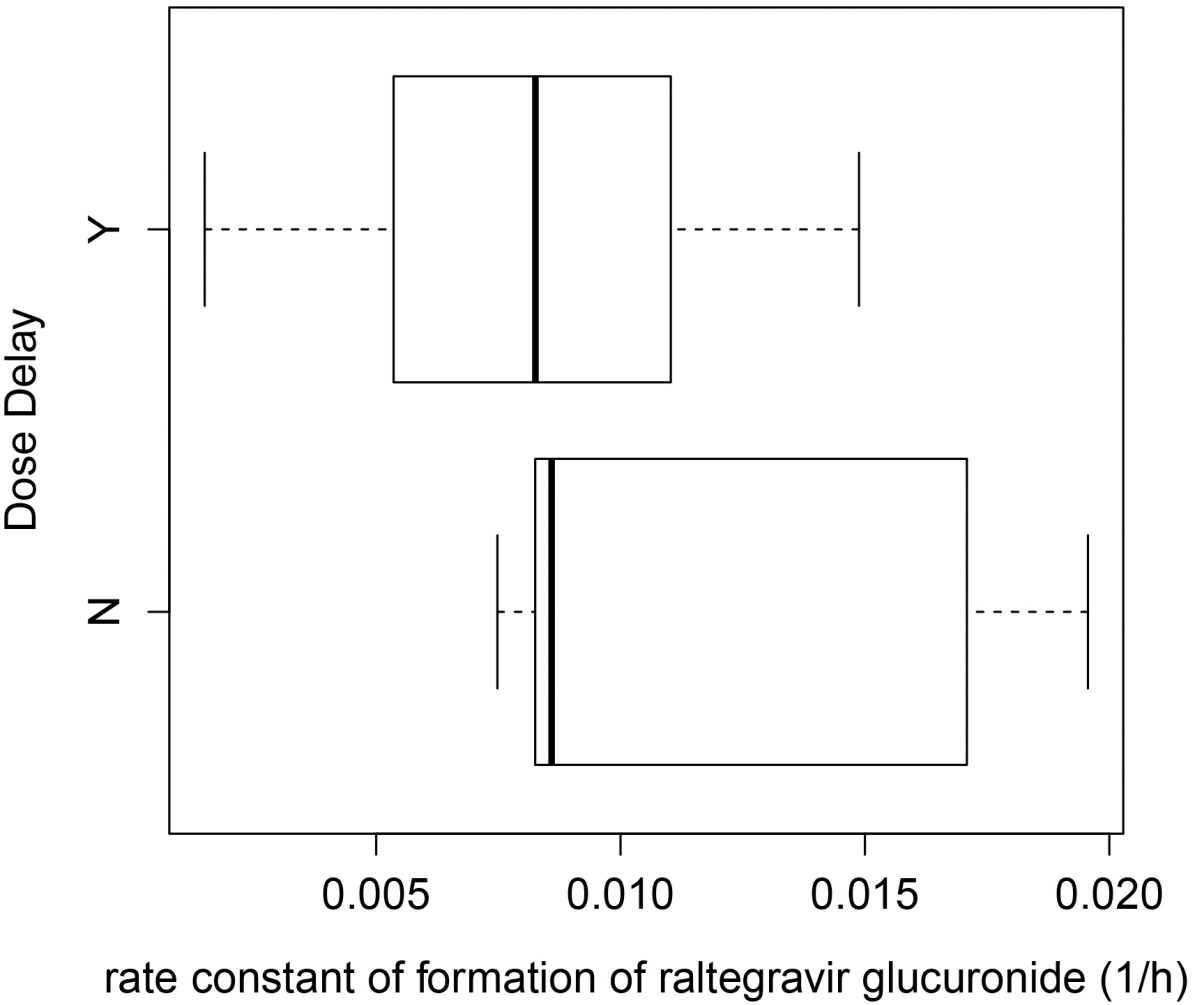
Box-plot showing the association between the rate constant of formation of raltegravir glucuronide from raltegravir (K_23_) and presence or absence of dose delay amongst the patients.

Significant correlation was found between nadir absolute neutrophil count (ANC) and rate constant of formation of raltegravir glucuronide from raltegravir (K_23_) across the 24 subjects (r = 0.598, P < 0.005) (Fig 3). In addition, analysis using the Wilcoxon rank sum test showed that K_23_ values in the presence or absence of dose delay amongst the subjects were not statistically significant (P = 0.0978) (Fig 4).

There was no significant correlation between 1-hydroxy-midazolam formation rate and nadir ANC (r = 0.071, p-value = 0.73). The relationship between the nadir ANC and the ratio of AUC(midazolam)/AUC(1-hydroxy-midazolam) was also not statistically significant (p-value = 0.515). In addition, there was no significant correlation between nadir ANC and bilirubin levels (data not shown).

### Genotype correlations

**Fig 5:**
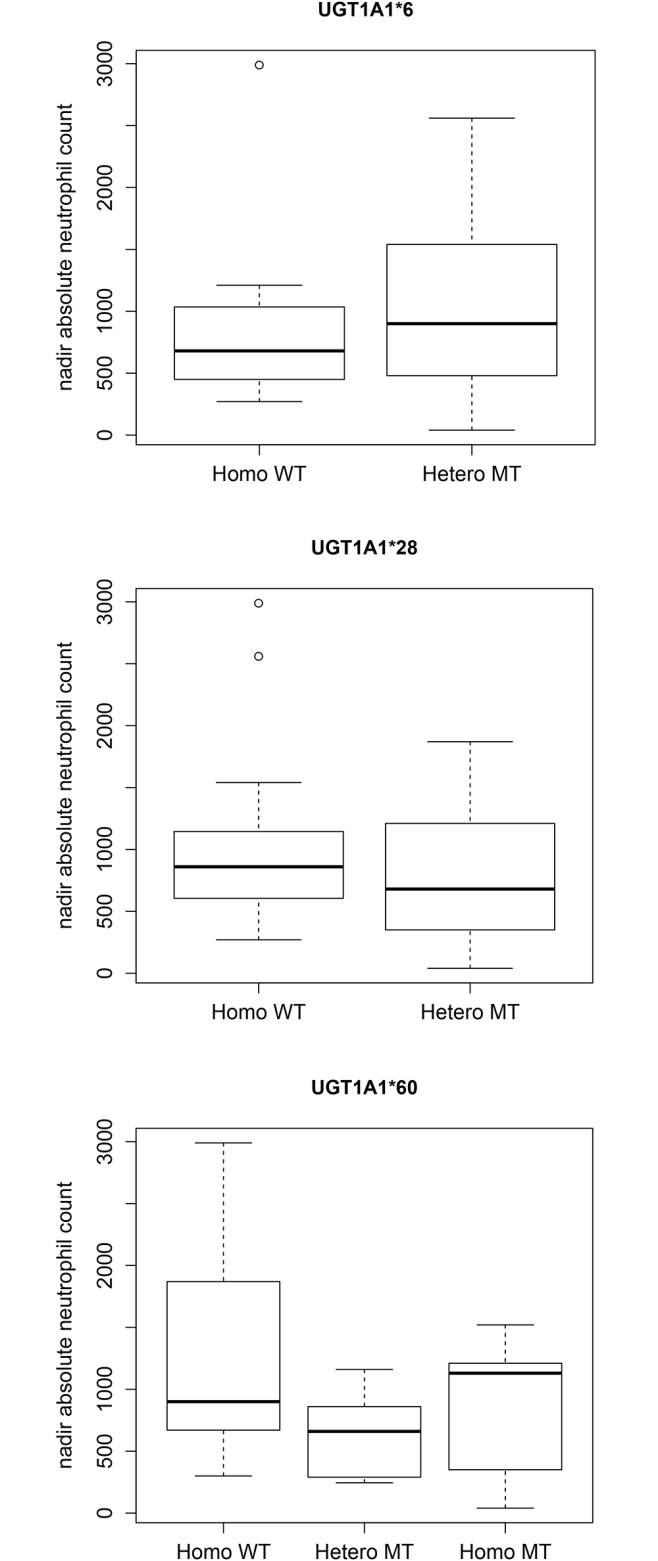
Box-plots showing the statistically insignificant associations between the nadir absolute neutrophil count and UGT1A1 genotypes.

No differences in toxicity (Fig 5) or SN-38 pharmacokinetics were observed in the UGT1A1 variants. Nonparametric statistical analyses showed that ANC values were not statistically significant between different allelic forms in the UGT1A1*6 (P = 0.551), UGT1A1*28 (P = 0.591) and UGT1A1*60 (P = 0.154) genotypes. The P value for SN-38 AUC was 0.106 between UGT1A1*28 wild type and heterozygous variant. The correlation (r) for UGT 1A1*6 versus nadir ANC is 0.382 (p-value = 0.0596). The r for UGT 1A1*6 versus K_23_ is 0.209 (p-value = 0.315). This suggests that the UGT 1A1*6 mutation was not a significant covariate for explaining the interindividual variability in raltegravir glucuronide formation. The r for the linear model of CYP 3A5 *3 versus ANC is 0.0925 (p-value = 0.66).

## Discussion

Metaanalysis of irinotecan pharmacogenetics studies show that there is an association between UGT1A1*28 homozygous genotype with neutropenia [22]. However, as the incidence of UGT1A1*28 is considerably lower in East Asians, genotyping for this variant may not be as clinically useful as in Western populations. In our study, there were no patients with UGT1A1*28 homozygotes or UGT1A1*28/UGT1A1*6 heterozygotes, though 2/3 of patients experienced grade 3–4 neutropenia in this study. Hence, we developed a new predictor for irinotecan pharmacokinetics and neutropenia, based on *in vivo* phenotyping of an individual's UGT1A1 activity. With respect to cytochrome P450s, raltegravir is a metabolically inert drug with little side effects. It is primarily metabolised by UGT1A1 to its glucuronide, similar to SN-38, the active metabolite of irinotecan. Despite the fact that raltegravir is orally administered, its clearance predicts the clearance of intravenously administered irinotecan and SN-38 well.

This method used modelling methods to estimate the formation rate constant of raltegravir glucuronide, therefore measuring the glucuronidation pathway directly. This approach proved to be successful as this constant correlated well with nadir ANC. Of the other parameters tested, only the AUC of the active metabolite SN-38 correlated with nadir ANC. However, individualizing the dose of irinotecan based on SN-38 is laborious and can only be done after at least the first dose has been administered, thus reducing the utility of this approach. Therefore in our study the use of raltegravir as a probe drug provided a way of individualizing irinotecan dose before the first dose of cytotoxic is actually administered.

Of note, neither the midazolam or irinotecan clearance nor UGT1A1 genetic variants correlated with nadir ANC. This could be due to the small sample size in our study. East Asians rarely harbour the UGT1A1*28 polymorphism, yet there is toxicity that is not explained. UGT1A1*6 seems to be less useful as a pharmacogenetic predictor of toxicity. Nevertheless, the ability of our approach to predict toxicity despite this small sample size limitation does give us optimism. We therefore propose that prospective studies be done to produce an algorithm to adjust doses of irinotecan based on raltegravir glucuronide formation. This could lead to reduced toxicity especially neutropenia, while maintaining outcomes. This approach could be useful to overcome the problem of differences between geographical regions of frequencies of UGT1A1 genotypes.

In conclusion, the current study supports the feasibility of using UGT1A1 activity to individualize irinotecan dose calculation. The application of this methodology could lead to reduced interindividual pharmacokinetic variability and reduced severe myelosuppression. In combination with genotyping, UGT1A1 phenotype determination should be explored further as a strategy to identify patients who are at risk for experiencing severe side effects following irinotecan administration, particularly in populations where UGT1A1*28 is less frequent.

## Supporting Information

S1 TREND Checklist TREND checklist. (PDF)

S1 Protocol Protocol for study, original. (DOC)
