# Supplementary material for: Phenotyping of UGT1A1 Activity Using Raltegravir Predicts Pharmacokinetics and Toxicity of Irinotecan in FOLFIRI
Source: PLoS One. 2016 Jan 25;11(1):e0147681. doi: 10.1371/journal.pone.0147681 (PMC4726617; doi:10.1371/journal.pone.0147681)
Supplement: S1 Protocol — (DOC) [file pone.0147681.s002.doc]

**Study of inter-individual and inter-ethnic differences in the degree of induction and inhibition of CYP3A and UGT1A1 in a multi-ethnic Asian population**

Protocol Version 1, 27 May 2010

Investigators:

Lee Soon Chin, MBBS, MRCP*

Lawrence S. Lee, MBBS, MRCP, PhD**

Goh Boon Cher, MBBS, MRCP*

Statistician:

Tai Bee-Choo, PhD**

* National University Hospital

Singapore

** National University of Singapore

Singapore

Study Site:

Investigational Medicine Unit

National University Hospital

Singapore

**1.0 INTRODUCTION**

1.1 Research Hypothesis

There is inter-individual and inter-ethnic variability in the degree of induction and/or inhibition of drug metabolizing enzymes such as cytochrome P450 subtype 3A4 (CYP3A4) and UDP-glucuronosyltransferase subtype 1A1 (UGT1A1) by commonly used concomitant medications. These differences may in part be due to genetic differences between individuals and ethnic groups.

1.2 Background

Inter-individual and inter-ethnic differences in drug handling exist, and are particularly pertinent for anti-cancer drugs that typically have narrow therapeutic indices. We recently reported ketoconazole inhibition of CYP3A4 to reduce docetaxel clearance and dose requirements by approximately 40% in a multi-ethnic Asian breast cancer cohort (n=31). However, despite inhibition of CYP3A4 by ketoconazole, inter-individual variability in docetaxel pharmacokinetics remained significant, suggesting that there are inter-individual variations to CYP3A4 inhibition by ketoconazole. Interestingly, Chinese had the lowest docetaxel clearance, highest docetaxel AUC and most neutropenia with ketoconazole modulation, followed by Malays and Indians (clearance 18.6±5.7, 23.7±9.3, 30.6±6.7L/h, p=0.048; AUC 4.2±1.4, 4.1±3.9, 2.4±0.5mg/L*h, p=0.048; grade 4 neutropenia 57%, 14%, 0%, p=0.024).[1]

These inter-ethnic differences were not observed in another cohort of multi-ethnic Asian breast cancer patients (n=63 Chinese, 28 Malay, 8 Indian) treated with standard dose docetaxel 75mg/m^2^m without ketoconazole, suggesting that inter-ethnic differences in ketoconazole inhibition of CYP3A4 exist.[2]

An exploratory genotyping analysis was performed on 4 orphan nuclear receptor genetic variants (*HNF4α*, *CAR*, *PXR*). In the ketoconazole-modulated cohort, patients who were homozygous wildtype for *HNF4αMet49Val* had more grade 4 neutropenia (75% vs 17%, AA vs AG/GG, p=0.003) compared to those with at least one variant allele; Chinese were more likely to be homozygous wildtype compared to Malays and Indians (50% vs 7% vs 0%, p=0.02). This variant had no impact on docetaxel PK or PD in patients treated with docetaxel without ketoconazole.[3]

We postulate that differences may also occur with induction of CYP3A with inducers such as rifampicin.

In addition, differences in inhibition and induction are likely to occur with other drug metabolism enzymes as well, such as UGT1A1.

1.3 Clinical Significance

The establishment of inter-ethnic differences in the degree of induction and/or inhibition of important drug metabolizing enzymes by common concomitant medications has significant clinical implications globally as drug interaction information on drug labels may have to be tailored for different ethnic populations. As CYP3A and UGT1A1 enzymes are involved in the metabolism of many commonly used anti-cancer agents, including chemotherapy (taxanes, vinca alkaloids, etoposide, cyclophosphamide, irinotecan, etoposide etc), endocrine therapy (tamoxifen, exemestane, etc), targeted therapy (gefinitib, imatinib, lapatinib, sunitinib etc), as well as supportive therapy (granisetron, aprepitant, etc), understanding these differences can help tailor therapy for the individual patient and for patients in different geographic locations.

**1.4 Study Rationale**

To study the above questions, we have chosen to use the probe drugs midazolam and raltegravir. Oral and intravenous midazolam is well-established as a specific probe for CYP3A [4]. Raltegravir is a new agent which is known to be metabolized by UGT1A1 [5]. These agents have been shown not to interact with each other [6] and thus can be used as a probe combination to measure the activity of CYP3A and UGT1A1 at the same time.

.

Ritonavir [7] and ketoconazole [8] are known potent inhibitors of CYP3A, and both have been used to boost anti-neoplastic drugs such as docetaxel [2, 9]. Ketoconazole is also an inhibitor of UGT1A1 [10]. Rifampicin is a broad-spectrum inducer and has been shown to increase CYP3A and UGT1A1 activity [11].

**2.0 STUDY OBJECTIVES**

2.1 Primary Objectives

To determine if there are inter-ethnic differences in the degree of induction and/or inhibition of CYP3A by common concomitant medications, using midazolam as a probe.

2.2 Secondary Objectives

**2.2.1.** To determine if there are inter-ethnic differences in the degree of induction and/or inhibition of UGT1A1 by common concomitant medications, using raltegravir as a probe.

**2.2.2.** To elucidate the genetic causes for differences in degree of enzyme induction and inhibition, with particular focus on genes that are known to regulate CYP3A and UGT1A1 function.

**2.2.3.** To determine if there are inter-ethnic differences in the degree of induction and/or inhibition of raltegravir intracellular accumulation and transporter expression by common concomitant medications

**3.0 STUDY DESIGN**

A randomized crossover 3 period (plus baseline) drug-drug interaction study in a healthy volunteer population comprising of Caucasians and 3 Asian ethnic groups, Chinese, Malay and Indian. Subjects will be recruited and administered known inducers or inhibitors of CYP3A enzymes followed by administration of probe drugs and pharmacokinetic profiling to evaluate enzyme function. Inter-ethnic comparison of drug pharmacokinetics will be performed.

Genotyping of candidate genes that are known to regulate the function of CYP3A (including NFI, RXR and PGRMC1) and UGT1A1 (including the *6 and *28 polymorphisms) will be carried out on samples collected from the healthy volunteer study, as well as from samples collected from breast cancer patients from two completed phase II studies comprising of Chinese, Malay and Indian patients, who were treated with standard dose (n=99) or reduced dose docetaxel modulated by ketoconazole (n=31).

**4.0 SELECTION AND ENROLLMENT OF SUBJECTS**

- 1. Inclusion Criteria

4.1.1 Subjects must have a Body Mass Index (BMI) of 18 to 30 kg/m^2^, inclusive. BMI = weight (kg)/ [height (m)]^2^ and weigh at least 50kg.

- - 1. Male or females, ages ≥ 21 to ≤ 65 years.
    2. Non-smoking or smoking no more than 10 cigarettes, or 2 cigars, or 2 pipes per day for at least 3 months prior to selection.
    3. Women of childbearing potential (WOCBP) must not be nursing or pregnant.
    4. All women of childbearing potential (have not been postmenopausal for at least 2 years nor undergone total hysterectomy) must have a negative serum β-HCG test performed at screening (within 24 hours before the start of study day 1), and use contraception for the duration of the trial and up to 2 weeks after administration of the last dose of drugs.

4.2 Exclusion Criteria

4.2.1 History or current evidence of any significant acute or chronic medical illness that, within the investigator’s discretion, would interfere with the conduct or interpretation of the study.

- - 1. Proven or suspected acute hepatitis at the time of study entry.
    2. Current or recent (within 3 months) gastrointestinal disease which would interfere with the conduct or interpretation of the study.
    3. Any major surgery within 8 weeks of enrollment. Any gastrointestinal surgery that could impact upon the absorption of study drug.
    4. Donation of blood or plasma within 56 days of screening.
    5. Inability to tolerate oral medication.
    6. Inability to tolerate venepuncture and/or absence of secure venous access.
    7. Inability to give informed consent voluntarily before the first trial-related activity.
    8. Known or suspected HIV infection or chronic HBV or HCV infection
    9. Known active drug or alcohol abuse, which in the opinion of the investigator makes study participation to completion unlikely.
    10. Any other significant medical, psychiatric and/or social issue as determined by the Investigator that would compromise subject’s safety and/or compliance with trial procedures.
    11. Subjects with AST, ALT or total bilirubin above the upper limit of normal.
    12. Haemoglobin < 10.9 g/dL, and platelet count < 125,000/mm^3^.
    13. Absolute Neutrophil Count (ANC) <1300/mm^3^
    14. Serum creatinine grade 1 or greater (≥ 1.1 x upper limit of laboratory normal range [ULN])
    15. Any other clinically significant screening lab abnormality (as determined by the investigator)
    16. Exposure to any investigational drug within 60 days of enrollment and throughout the study.
    17. Any previous clinically significant allergy or hypersensitivity or intolerance to midazolam, raltegravir, ritonavir, ketoconazole and rifampicin, or any other ingredient of the tablets or capsules.
    18. Use of any agent, within 2 weeks of dosing, that is known to induce or inhibit drug‑metabolizing enzymes.
    19. Use of concomitant medication, including investigational, prescription, and any over-the-counter drugs and dietary supplements with the following exceptions, aspirin, paracetamol, diphenhydramine, daily multivitamins, mineral supplements and hormonal oral contraceptives. Concomitant medication other than those listed above must have been discontinued within 14 days of study entry.

**4.3 Prohibitions and Restrictions**

- - 1. Subjects may not consume beverages containing alcohol or quinine (e.g., tonic, bitter lemon, bitter alcoholic beverages containing quinine) between 24 hours before the first intake of trial medication until 24 hours after the last intake of trial medication. Grapefruit, grapefruit juice, orange and orange juice are not allowed between 7 days before the first intake of trial medication until 24 hours after the last intake of trial medication.
    2. Subjects will not significantly change their normal level of exercise and will refrain from strenuous activities during the duration of the trial.
    3. If a subject has had a recent febrile illness, the first intake of trial medication should be postponed until body temperature is normal for at least 72 hours.
    4. Subjects will be advised not to donate blood for at least 90 days after the last intake of trial medication.
    5. Extreme exposure to the sun or sunbathing should be avoided, as well as the use of tanning devices (e.g., sunbed, solarium) and topical tanning products from screening until the last trial-related visit.
    6. Smoking subjects will not change their stable smoking habits during the duration of the trial.

4.4 Study Enrollment Procedures

Prior to implementation of this protocol, the protocol and consent form will be approved by the institutional review board (IRB). Once a candidate for study entry has been identified, details will be carefully discussed with the subject. The subject will be asked to read and sign the consent form that was approved by the IRB prior to any study procedures. Subjects meeting eligibility criteria will be enrolled in the study. We estimate that approximately 18 will be enrolled in each of the 4 ethnic groups order to obtain 12 evaluable subjects. Toxicity-related attrition and drop-outs may occur, and accrual will continue as necessary to ensure that 12 evaluable subjects complete the study.

**5.0 STUDY TREATMENT**

At baseline, healthy volunteers will take the probe drugs oral (1.5 mg) and intravenous (0.75 mg) midazolam and oral raltegravir (400 mg), and a pharmacokinetic profile performed. There will be a washout period of at least 3 days.

In Period 1, healthy volunteers will be randomized to take the inhibitor drug ritonavir 100 mg BD or ketoconazole 200 mg BD for 3 days, and then receive the probe drugs and pharmacokinetic profile. There will be a washout period of at least 3 days.

In Period 2, healthy volunteers will take the other inhibitor drug, ketoconazole 200 mg BD or ritonavir 100 mg BD for 3 days, and then receive the probe drugs and pharmacokinetic profile. There will be a washout period of at least 3 days.

In Period 3, healthy volunteers will receive rifampicin 600 mg every night for 14 days. Subjects will take the probe drugs on days 7 and 14 and have pharmacokinetic profiles done at each time.

**5.1 Administration of Study Drugs**

**5.1.1 Timing and specifics of Dosing**

Morning medications should be taken between 8 and 9 AM. Evening medications should be taken between 8 and 9 PM. On pharmacokinetic profile mornings, medications will be administered by study personnel fasted with 240 ml of water.

**5.1.1.1 Ritonavir (RTV, Norvir®)**

RTV will be administered as one 100 mg capsules (100 mg) orally every 12 hours within 30 minutes after a meal or snack. RTV capsules are soft gelatin white capsules imprinted with the corporate logo, 100 and the Abbott Code DS. RTV will be purchased by the study site.

**5.1.1.2 Ketoconazole (KCZ , Nizoral®)**

KCZ will be administered as one 200 mg tablet orally once daily within 30 minutes after a meal or snack. KCZ tablets will be purchased by the study site.

**5.1.1.3 Rifampicin (RIF, Rifaren®)**

RIF will be administered as two 300 mg capsules orally nightly before a meal. RIF capsules will be purchased by the study site.

**5.1.1.4 Midazolam (MDZ, Dormicum®)**

Intravenous midazolam is stored in 5 mg vials in 1 ml of solvent.

Oral midazolam syrup will be obtained and given fasted with water.

**5.1.1.5 Raltegravir (RAL, Isentress®)**

RAL will be administered as one 400 mg tablet fasted with water.

5.2 Handling and Dispensing of Investigational Products

Investigators will be responsible for assuring that the storage and handling of the investigational product is adequate for the duration of the trial.

RTV capsules should be stored in the refrigerator at 36F-46°F (2-8°C) until dispensed. May store capsules out of refrigerator at less than 77°F (2°C) for up to 30 days. Protect from light and excessive heat.

RIF tablets should be stored at room temperature (below 25 °C) and protected from light and moisture.

RAL tablets should be stored at 20-25°C (68-77°F) with excursions permitted to 15-30°C (59-86°F).

Investigational products will be stored in a secure area according to local regulations. It is the responsibility of the Investigator to ensure that investigational products are only dispensed to study subjects.

5.2.1 Return and Destruction of Investigational Product

Upon completion or termination of the study, all unused and/or partially used DRV must be destroyed at the site. Documentation of the remaining inventory and the method of destruction will be documented by a certificate of destruction.

**5.3 Concomitant Medications**

**5.3.1 Permitted Medications**

Paracetamol (up to 1 g 6h prn) as an analgesic

Cetirizine (5-10 mg OD prn) or levocetirizine (5mg OD prn) as an antihistamine. However, the use of diphenhydramine cannot be used concomitantly with study drugs during the days PK data are being collected.

Diphenhydramine (25 mg prn) as a sleeping aid. However, the use of diphenhydramine cannot be used concomitantly with study drugs during the days PK data are being collected.

Imodium (loperamide) tablets taken as 4 mg po for the initial dose and then 2 mg po Q6 hours prn is permitted to manage diarrhea. On PK days no doses may be given until 4 hours after study drugs are given.

Prochlorperazine 10 mg taken 1 hour before dosing of and drugs is permitted if needed for nausea and may also be used up to twice a day if needed for nausea associated with study drug administration. Prochlorperazine may be used pre-treatment 1 hour before study medications on PK days if needed. No further doses may be given until 4 hours after study drugs are given on PK days.

Use of these agents must be recorded on the appropriate case report form (CRF).

**5.3.2 Prohibited Medications**

The use of all investigational, prescription, and over-the-counter medications aside from those listed in section 5.2.1 is prohibited while subjects are on study. Also, the use of any herbal remedies, e.g., St. John’s wort, is not allowed. The use of specific medications may be considered by the study team on a case-by-case basis. Restrictions on medications taken prior to enrollment in the study are described in Section 4.0. Medications taken within 2 weeks of study enrollment must be recorded on the CRF. No prohibited concomitant medications or therapies, prescriptions, over-the-counter or herbal preparations, are to be administered during study participation unless they are prescribed by the Investigator for treatment of specific clinical events at which time subjects will be removed from the study. Any concomitant therapies must be recorded on the CRF.

**5.3.3 Dietary Restrictions**

Due to potential interactions between protease inhibitors and grapefruit juice and oranges, all subjects must refrain from consuming grapefruits, oranges or any product containing grapefruit or oranges for 7 days prior to study entry, while on study and 24 hours after the last study dose. Consumption of alcohol is prohibited 24 hours prior to the first dose of study drug, during the study period and 24 hours after the last dose of study drug.

## 5.4 Adherence Assessment

**5.4.1 Adherence Assessment**

Subjects will complete a daily Medication Diary which will provide a self-reported measure of study drug adherence. Subjects will be asked to record the date, time, and amount of study medication taken. Subjects will be asked to check off in the Medication Diary if study medication was taken within 30 minutes following a light meal or snack. They will also be required to record any other medications (including OTCs, vitamins, or herbal preparations as they are disallowed by protocol) taken during the study period in the diary provided. Subjects will also be queried at each visit about medications taken (including OTCs, vitamins, or herbal preparations). This diary will be reviewed with the study subject by study personnel at each study visit.

All doses on PK evaluation days are administered under direct supervision by study personnel. All doses and times will be recorded in the CRF.

- - 1. **Pill Counts**

A known number of pills will be dispensed during the study on specified days at the beginning of each regimen period. Subjects will be instructed to bring their pill containers with them to each clinic visit. Those pills remaining at the next visit will be counted to establish an estimate of inter-visit adherence.

# 6.0 CLINICAL AND LABORATORY EVALUATIONS

**6.1 Special Instructions and Definitions of Evaluations**

**6.1.1 Clinical Assessments**

Vital Signs

Vital signs will include: body temperature, respiratory rate, blood pressure and heart rate (supine). These measurements will be obtained after the subject has rested for at least 5 minutes.

Physical Examinations

Complete physical examinations or targeted physical examinations will be performed by a physician or a physician’s assistant as specified during the study (Sec 7.0). Targeted physical examinations are defined as those that include examinations focused specifically on an organ system(s) of interest in the evaluation of a perceived change in the clinical status of a patient or in the evaluation of an adverse event. Subjects will be queried for adverse events in an open-ended manner.

***Laboratory Test Assessments***

Blood samples will be obtained by venepuncture or via an indwelling catheter and urine samples will be obtained for clinical laboratory evaluations as specified below. The clinical laboratory performing the analyses of all scheduled laboratory tests will provide reference ranges for these tests. The following clinical laboratory tests will be performed:

| **Haematology** (screen, day 1, 7, 14, 30, and follow up) |
| --- |
| Haemoglobin |
| Haematocrit |
| Total leukocyte count, including differential |
| Platelet count |
| **Serum Chemistry** (screen, day 1, 7, 14, 30, and follow up) |
| CK (screen only and if symptomatic) |
| Alanine aminotransferase (ALT) |
| Aspartate aminotransferase (AST) |
| Total bilirubin |
| Alkaline phosphatase |
| Creatinine |
| Blood urea nitrogen (BUN) |
| Blood glucose |
| Total protein |
| Albumin |
| Sodium |
| Potassium |
| Chloride |
| Calcium |
| Lipase (screen only and if symptomatic) |
| Pancreatic amylase (screen only and if symptomatic) |
| **Urinalysis** (screen only) |
| Specific gravity |
| pH |
| Protein |
| Glucose |
| Ketones |
| Blood |
| Leukocyte esterase |
| Nitrites |
| Microscopic examination of the sediment if blood, protein, leukocytes or nitrites are positive on the dipstick |
| **Other Analyses** |
| Genotyping for CYP3A4/5 and UGT1A1 (screening only) |
|  |
| HIV-serology (screening only) |
| Urine pregnancy test (screening only) (Women of Child Bearing Potential only) |
| Hepatitis B Surface Antigen (screening only) |
| Hepatitis C Antibody (Qualitative PCR to be run if antibody test is positive) (screening only) |

**6.1.2** **Pharmacokinetic Evaluations**

**6.1.2.1 Pharmacokinetic parameters**

Pharmacokinetic parameters of MDZ and RAL will be derived from plasma concentration versus time data. Samples will be drawn on specified study days (Sec 7.0). The PK parameters to be assessed include:

| Cmax | Maximum observed plasma or serum concentration |
| --- | --- |
| Tmax | Time of maximum observed plasma or serum concentration |
| AUC-(TAU) | Area under the plasma concentration-time curve in two dosing interval over 12 hours. |
| Cmin/  C_tau_ | Trough plasma concentrations at 12 hours. |

Individual subject PK parameter values will be derived by non-compartmental methods using a validated PK program (WinNonlin).

6.1.2.2 Pharmacokinetics: Blood Collection and Processing

Blood samples for the measurement of midazolam (MDZ) and raltegravir (RAL) (3 mL each timepoint; concentrations will be determined from the same plasma sample) will be collected from an indwelling catheter or by direct venipuncture into an Lithium heparin-containing Vacutainer tube. Approximately 3 mL of blood will be collected from each subject for pharmacokinetic purposes. If a catheter is used for blood collection then prior to blood sampling the fluid in the catheter will be completely withdrawn at each sampling time and discarded. Immediately after collection, each tube will be gently inverted a few times to completely mix the anticoagulant and then placed in wet ice. See additional details in Appendix 1.

Table 6.1.2.2: Summary of drug concentration measurements

| **Analyte** | **Study day** | **Nominal sample time in hours (relative to dosing)** | **Blood sample volume (mL)** |
| --- | --- | --- | --- |
| MDZ, RAL | Day 1 | 0 (pre-oral dose), 0.25, 0.5, 0.75, 1, 1.5, 2, 2.5, 3, 3.5, 4 (pre-IV dose), 4.25, 4.5, 4.75, 5, 5.5, 6, 7, 8, 10, 12 | 63 |
| MDZ, RAL, (RTV or KCZ) | Day 7 (+/- 3 days) | 0 (pre-oral dose), 0.25, 0.5, 0.75, 1, 1.5, 2, 2.5, 3, 3.5, 4 (pre-IV dose), 4.25, 4.5, 4.75, 5, 5.5, 6, 7, 8, 10, 12 | 63 |
| MDZ, RAL, (KCZ or RTV) | Day 14 (+/- 3 days) | 0 (pre-oral dose), 0.25, 0.5, 0.75, 1, 1.5, 2, 2.5, 3, 3.5, 4 (pre-IV dose), 4.25, 4.5, 4.75, 5, 5.5, 6, 7, 8, 10, 12 | 63 |
| MDZ, RAL, (RIF) | Day 30 (+/- 3 days) | 0 (pre-oral dose), 0.25, 0.5, 0.75, 1, 1.5, 2, 2.5, 3, 3.5, 4 (pre-IV dose), 4.25, 4.5, 4.75, 5, 5.5, 6, 7, 8, 10, 12 | 63 |

**6.1.2.3 Missed doses within 72 hours of Inpatient PK Sampling**

PK sampling can not be performed if any dose is missed within the 72-hour period prior to a PK session. When that occurs subjects may be allowed to take an additional 24 hours of study medications prior to obtaining PK sampling.

**6.1.3** **Urine collection**

Urine will be collected at predose, 0-4, 4-8 and 8-12 hours postdose for measurement of drugs and metabolites.

The volume of urine collected will be measured precisely. 2 aliquots of 10 mL will be stored at -80C until further analyses.

**6.1.4** **Drug analyses**

Concentrations of midazolam and the metabolite 1-OH-midazolam in plasma will be quantified using a validated LC/MS method.

Quantitation of raltegravir and its glucuronide in plasma and urine will also be quantified using a validated LC/MS method.

Quantitation of inhibitor or inducer drugs can also be performed later if deemed necessary.

**6.1.5** **Transporter expression**

An additional 10 mL of blood will be drawn on days 1, 7, 14 and 30 to measure expression of transporters on peripheral blood mononuclear cells. This blood can be drawn at any time of the day.

**6.1.6** **Intracellular drug concentrations**

An additional 8 mL of blood will be drawn on days 1, 7, 14 and 30 in CPT tubes to isolate peripheral blood mononuclear cells. Cells will be counted and stored based on an already established method used for other raltegravir trials. This blood should be drawn at the 3 hour timepoint (anticipated peak raltegravir concentration).

**7.0 STUDY PROCEDURES**

**7.1 Screening (Within 60 days prior to Study Entry)**

Details of the study will be carefully discussed with the subject and the subject will be asked to read and sign informed consent prior to determining if the subject meets the entry criteria for the study.

A medical history will be obtained and a complete physical examination including vital signs, height, and weight will be performed.

Laboratory evaluations as described in section 6.0 will be obtained

An additional blood sample for genotyping will also be drawn (3ml of blood in one Lithium heparin tube).

Subjects will be chosen to ensure adequate representation of different genotypes.

18 subjects from each of the 4 major races will be screened to allow 12 subjects to be enrolled.

- 1. **Baseline**
     1. **Day 0**

Subjects meeting inclusion/exclusion criteria will report to the clinic for baseline evaluations the night before they are to begin study drug administration.

Subjects will be queried for any changes in their health, physical or smoking activity, and non-study drug medication consumption (including consumption of restricted foods and beverages listed) since the screening period.

Notify subjects that they are not to change their smoking or exercise habits during the course of the study period. Notify subjects that they may not consume any alcohol or restricted foods and beverages during the course of the study.

Subjects will be fasted after midnight except water.

- - 1. **Day 1**

A targeted physical examination and vital signs will be performed.

Laboratory evaluations as described in section 6.0 will be obtained.

An indwelling catheter will be inserted into an arm vein for drawing serial blood samples.

Subjects will be dispensed the probe medications regimen (MDZ 1.5 mg PO then 0.75 mg IV 4 hours later, and RAL 400 mg). Oral medications will be given fasted with water.

Subjects will then be NPO except water until the 5 hour PK sample is collected.

Blood for pharmacokinetic analysis will be collected at 0 (pre-oral dose), 0.25, 0.5, 0.75, 1, 1.5, 2, 2.5, 3, 3.5, 4 (pre-IV dose), 4.25, 4.5, 4.75, 5, 5.5, 6, 7, 8, 10, 12 hours post oral dosage.

After the 12 hours sampling, subjects can go home.

**7.3 Period I (Study Days 4 - 7, up to 3 days window period allowed after washout of at least 3 days)**

**7.3.1** **Day 4 (outpatient visit) or up to 3 days later**

Subjects will come to the clinic to receive their first inhibitor medication, RTV 100 mg BD or KCZ 200 mg BD.

Subjects will be given a medication diary to record the times and amounts of all doses of study medications taken and any other non-study medications taken. They will be instructed to bring this diary to every visit. Subjects will be instructed that they may not start any new medications unless approved by a member of the study team.

**7.3.2 Inpatient Pharmacokinetic Evaluation (Day 6-7)**

**Day 6:**

The subjects will be admitted to the Inpatient Unit at night, they will be NPO after midnight except for water. The nurse will check that subjects take their evening dose of the first inhibitor.

**Day 7:**

A targeted physical examination and vital signs will be performed.

Laboratory evaluations as described in section 6.0 will be obtained.

An indwelling catheter will be inserted into an arm vein for drawing serial blood samples.

Subjects will be dispensed the probe medications regimen (MDZ 1.5 mg PO then 0.75 mg IV 4 hours later, and RAL 400 mg). Oral medications will be given fasted with water.

Subjects will then be NPO except water until the 5 hour PK sample is collected.

Blood for pharmacokinetic analysis will be collected at 0 (pre-oral dose), 0.25, 0.5, 0.75, 1, 1.5, 2, 2.5, 3, 3.5, 4 (pre-IV dose), 4.25, 4.5, 4.75, 5, 5.5, 6, 7, 8, 10, 12 hours post oral dosage.

After the 12 hours sampling, subjects can go home.

**7.4 Period 2 (Study Days 11 - 14, up to 3 days window period allowed after washout of at least 3 days)**

**7.4.1** **Day 11 (outpatient visit) or up to 3 days later**

Subjects will come to the clinic to receive their second inhibitor medication, KCZ 200 mg BD or RTV 100 mg BD.

Subjects will be given a medication diary to record the times and amounts of all doses of study medications taken and any other non-study medications taken. They will be instructed to bring this diary to every visit. Subjects will be instructed that they may not start any new medications unless approved by a member of the study team.

**7.4.2 Inpatient Pharmacokinetic Evaluation (Day 13-14)**

**Day 13:**

The subjects will be admitted to the Inpatient Unit at night, they will be NPO after midnight except for water. The nurse will check that subjects take their evening dose of the second inhibitor.

**Day 14:**

A targeted physical examination and vital signs will be performed.

Laboratory evaluations as described in section 6.0 will be obtained.

An indwelling catheter will be inserted into an arm vein for drawing serial blood samples.

Subjects will be dispensed the probe medications regimen (MDZ 1.5 mg PO then 0.75 mg IV 4 hours later, and RAL 400 mg). Oral medications will be given fasted with water.

Subjects will then be NPO except water until the 5 hour PK sample is collected.

Blood for pharmacokinetic analysis will be collected at 0 (pre-oral dose), 0.25, 0.5, 0.75, 1, 1.5, 2, 2.5, 3, 3.5, 4 (pre-IV dose), 4.25, 4.5, 4.75, 5, 5.5, 6, 7, 8, 10, 12 hours post oral dosage.

After the 12 hours sampling, subjects can go home

**7.5 Period 3 (Study Days 17 - 30, up to 3 days window period allowed after washout of at least 3 days)**

**7.4.1** **Day 17 (outpatient visit) or up to 3 days later**

Subjects will come to the clinic to receive their inducer medication, RIF 600 mg ON.

Subjects will be given a medication diary to record the times and amounts of all doses of study medications taken and any other non-study medications taken. They will be instructed to bring this diary to every visit. Subjects will be instructed that they may not start any new medications unless approved by a member of the study team.

**7.4.2 Inpatient Pharmacokinetic Evaluation 1 (Day 29-30)**

**Day 29:**

The subjects will be admitted to the Inpatient Unit at night, they will be NPO after midnight except for water. The nurse will check that subjects take their evening dose of the inducer.

**Day 30:**

A targeted physical examination and vital signs will be performed.

Laboratory evaluations as described in section 6.0 will be obtained.

An indwelling catheter will be inserted into an arm vein for drawing serial blood samples.

Subjects will be dispensed the probe medications regimen (MDZ 1.5 mg PO then 0.75 mg IV 4 hours later, and RAL 400 mg). Oral medications will be given fasted with water.

Subjects will then be NPO except water until the 5 hour PK sample is collected.

Blood for pharmacokinetic analysis will be collected at 0 (pre-oral dose), 0.25, 0.5, 0.75, 1, 1.5, 2, 2.5, 3, 3.5, 4 (pre-IV dose), 4.25, 4.5, 4.75, 5, 5.5, 6, 7, 8, 10, 12 hours post oral dosage.

After the 12 hours sampling, subjects can go home.

**7.6 Follow-up visit (Day 37 +/- 4 days)**

Subjects will be asked to return for a follow-up visit one week from the date of discharge.

Vital signs and safety labs will be collected. Subjects will be queried for any adverse events and use of any concomitant medications since discharge.

Subjects may be asked to return for additional follow up of any abnormalities deemed important by the investigator. These abnormalities will be followed until resolution or stabilization. This follow up may include repeating study assays such as transporter assays, if the subject agrees to have the extra blood taken.

1. WITHDRAWAL OF SUBJECTS FROM STUDY

Subjects must be discontinued from study therapy and withdrawn from the study for the following reasons:

- Request by the subject to be withdrawn.
- Drug-related toxicity as outlined in Section 11.0 and Appendix II
- Any clinical adverse event, laboratory abnormality or intercurrent illness which, in the opinion of the investigator, indicates that continued treatment with study therapy and participation in the trial is not in the best interest of the subject, even if not addressed in the toxicity management section of the protocol.
- Use of study-prohibited medications
- Pregnancy
- Termination of the study by sponsor.
- Imprisonment or the compulsory detention for treatment of either a psychiatric or physical (e.g., infectious disease) illness.
- Subject judged by the investigator to be at significant risk of failing to comply with the provisions of the protocol as to cause harm to self or seriously interfere with the validity of the study results.
  - Any more than one 24 hour period of medication non-compliance within 72 hours of inpatient PK sampling.

**9.0 STATISTICAL METHODOLOGY**

9.1 Data Set Descriptions

All available data from subjects with complete pharmacokinetic data available will be included in the pharmacokinetic data set.

All subjects who receive study drug will be included in the safety data set.

9.2 Sample Size Determination

18 subjects will provide 80% power to detect a difference between the groups with an alpha of 5%. To allow for attrition, 18 subjects will be enrolled. This power calculation is based on doing a one-way ANOVA analysis, assuming a mean clearance of 18.6 for Chinese, 23.7 for Malay and 30.6 for Indian, with a common standard deviation of 9.

9.3 Pharmacokinetic Parameter Statistical Analyses

### 9.3.1 Primary analyses

The primary aim of this trial is to obtain estimates for the effect of the 2 inhibitors and inducer on oral and intravenous clearance of midazolam.

Analysis will be carried out using WinNonlin (Version 5, Pharsight, Cary, NC). The linear-log trapezoidal method will be used to calculate AUC 0-4 and AUC 0-12. For the noncompartmental evaluation, the AUC attributable to the oral midazolam dose will be defined as the AUC after oral administration until the point of time just before intravenous administration, plus the AUC from the point of time when the concentration just before intravenous administration was reached again after intravenous administration to infinity. The point of time when the concentration just before intravenous administration was reached again after intravenous administration will be obtained by log-linear extrapolation from the two adjacent points of the concentration versus time profile. The AUC attributable to the intravenous dose will be calculated as overall AUC minus the AUC attributable to the oral dose.

Clearance of i.v. midazolam will be calculated using the formula: CL_iv_ = IV dose/AUC_0-inf_ . Hepatic availability of i.v. administered midazolam will be calculated as Fhepatic = 1-CL_iv_/liver blood flow, and the liver blood flow will be estimated as 25.3 ml/kg body weight/min. Intestinal availability of oral midazolam will be calculated as F_intestinal_ = F_oral_/F_hepatic_, whereas absolute bioavailability following oral administration of midazolam will be calculated as F_oral_ = AUC_oral_/AUC_iv_ corrected for dose differences.

Statistical comparisons will be performed after logarithmic transformation. PK parameters will be reported as geometric mean (GM) with 90% confidence intervals (CI). Assuming normal distribution, comparisons of MDZ parameters with and without inhibitor/inducer will use the paired t-test.

### 9.3.2 Secondary pharmacokinetic analyses

The secondary aims of this trial are to obtain estimates for the effect of inhibitors and inducer on the clearance and intracellular accumulation of raltegravir, and the influence of CYP3A and UGT1A1 genotypes on the above effects. For the secondary pharmacokinetic analyses, the parameters will be assessed statistically using the same methods as described for the primary endpoints.

### 9.3.3 Secondary safety analyses

For the safety analyses, descriptive statistics will be used to summarize and evaluate adverse events and clinically relevant laboratories. All subjects who received at least one dose of study medication will be included in the safety analyses. Standard statistical measurements (e.g., mean, median, standard deviation, quartiles, range and number of non-missing values) or frequency tables will be calculated where appropriate.

**10.0 RISKS**

**10.1 Ritonavir (RTV)**

The most common adverse events experienced by HIV-infected patients on RTV 400 mg BD were asthenia, gastrointestinal and neurological disturbances including nausea, diarrhea, vomiting, anorexia, abdominal pain, taste perversion, and circumoral and peripheral paresthesias [12]. RTV is also associated with ALT or/and AST elevation, triglyceride elevation and cholesterol elevation.

When RTV is administered to healthy volunteers the most common adverse events are diarrhea, headache, nausea, and vomiting. We expect that the incidence of adverse events will be low with the dose of 100 mg BD for just 3 days.

**10.2 Ketoconazole (KCZ)**

The most frequent adverse reactions with KCZ were nausea and/or vomiting in approximately 3%, abdominal pain in 1.2%, pruritus in 1.5%, and the following in less than 1% of the patients: headache, dizziness, somnolence, fever and chills, photophobia, diarrhea, gynecomastia, impotence, thrombocytopenia, leucopenia and hemolytic anemia. Oligospermia has been reported in investigational studies with the drug at dosages above those currently approved.

Most of these reactions were mild and transient and rarely required discontinuation of ketoconazole tablets. Rare occurrences of hepatic dysfunction have occurred. Neuropsychiatric disturbances, including suicidal tendencies and severe depression, have occurred rarely.

**10.3 Rifampicin (RIF)**

Adverse events due to RIF include cutaneous reactions which are mild and self-limiting and do not appear to be hypersensitivity reactions. Typically they consist of flushing and itching with or without a rash. Urticaria and more serious hypersensitivity cutaneous reactions have occurred but are uncommon. Gastrointestinal reactions consist of anorexia, nausea, vomiting, abdominal discomfort, and diarrhoea. Hepatitis can be caused by rifampicin and liver function tests should be monitored.

Reactions usually occurring with intermittent dosage regimens and probably of immunological origin include:

- 'Flu Syndrome' consisting of episodes of fever, chills, headache, dizziness, and bone pain appearing most commonly during the 3rd to the 6th monthly of therapy. The frequency of the syndrome varies but may occur in up to 50 % of patients given once-weekly regimens with a dose of rifampicin of 25 mg/kg or more.
- Shortness of breath and wheezing
- Decrease in blood pressure and shock
- Anaphylaxis
- Acute haemolytic anaemia
- Acute renal failure usually due to acute tubular necrosis or acute interstitial nephritis

**10.4 Raltegravir (RAL)**

In previous healthy volunteer studies, raltegravir was generally well tolerated. No serious clinical or laboratory adverse experiences were reported and no subjects discontinued because of an adverse experience. Of the 93 non-serious clinical adverse experiences reported by 40 subjects, 25 were considered by the investigator to be possibly related to study drug. The most common drug-related adverse experiences were headache and fatigue [13]. All adverse experiences reported were transient and mild to moderate in intensity. Laboratory adverse experiences were not observed, and neither were consistent treatment-related changes in laboratory, vital signs, or electrocardiogram safety parameters.

**10.5 Midazolam (MDZ)**

Adverse reactions in the dose given in this study are expected to be low in incidence and severity. Reported adverse reactions include: Hiccups, drowsiness, hypotension, bradycardia, ataxia, amnesia, dizziness, headache, nausea, vomiting, laryngospasm, hallucination, respiratory depression, apnoea, rash.

Treatment of midazolam toxicity

Treatment is supportive, and rarely is mechanical ventilation required. The specific antidote for overdosing is flumazenil. The dose of flumazenil is 0.2 mg intravenously over 15 seconds.

**10.6 Unknown Risks**

Although all of the medications being evaluated in this study have been approved by the FDA, there is potential for additional, increased or unknown risk when they are given together. However, based on the safety profiles of these medications in clinical trials and their wide therapeutic index it is unlikely that an unknown risk will arise from co-administration.

**10.7 Blood drawing and Intravenous Access**

Unless the subject has a severe bleeding disorder, the risk and discomforts of these procedures are slight. The vein may be damaged by the needle and cause minor bleeding and/or a temporary black and blue spot; rarely fainting, phlebitis or cellutitis may occur. In some subjects, it may be impossible to place or maintain an IV line in place for PK assessments. For these subjects, blood may be obtained using standard blood drawing techniques. The total amount of blood collected during the study will be about 350 cc. This is less than the amount of blood donated at a standard Red Cross blood donation.

## 11.0 CRITERIA FOR TOXICITY MANAGEMENT AND STUDY DRUG DISCONTINUATION.

**11.1 General**

Subjects will be instructed to notify the study team immediately if they develop any adverse signs or symptoms. They will be closely monitored for signs and symptoms of hepatic, gastrointestinal and renal side effects.

AEs will be checked at every visit and reported from signing the consent form onwards until the last trial related visit. Severity and drug relationship of AEs towards darunavir, ritonavir, and efavirenz will be recorded.

Special attention will be paid to those subjects who discontinue the trial for an AE, or who experience a severe AE (at least grade 3), or a SAE. In case of dropout, subjects will come, if consent not withdrawn, for an early termination visit at time of discontinuation.

If subjects develop AEs or unexpected blood results, they and other subjects may be recalled for safety visits to repeat the blood tests.

**Grade 1**

Subjects who develop a grade 1 AE or toxicity may continue the intake of trial medication.

**Grade 2**

Subjects who develop a grade 2 AE or toxicity (except cutaneous reaction/rash, see below) may continue the intake of trial medication or be withdrawn based on the investigator’s clinical judgment.

**Grade 3 or 4**

Any Grade 3 or 4 AE, toxicity or laboratory result confirmed by repeat analysis as defined in Appendix II will result in termination of all study drugs and followed as appropriate until resolution.

For subjects experiencing specific AEs, toxicity management should be done as described below. Any clinically significant abnormalities persisting at the end of the study will be followed by the investigator until resolution or until reaching a clinically stable endpoint.

**11.2 Specific Toxicities**

For all toxicities that require study therapy to be permanently discontinued, relevant clinical and laboratory tests will be repeated as needed until there is final resolution or stabilization of the toxicity.

For subjects reporting rash, allergic reaction, AST/ALT elevations, clinical hepatitis, renal complications, nausea, or diarrhea, the following should be done.

**ACUTE SYSTEMIC ALLERGIC REACTION**

**Grade 1 (Localized Urticaria [Wheals] With no Medical Intervention Indicated)**

Subjects may continue the intake of trial medication.

Cetirizine, levocetirizine, topical corticosteroids or antipruritic agents may be prescribed.

Subjects should be advised to contact the investigator immediately if there is any worsening of the acute systemic allergic reaction.

Grade 2 (Localized Urticaria With Medical Intervention Indicated, or Mild Angioedema With no Medical Intervention Indicated)

Subjects may continue the intake of trial medication.

Cetirizine, levocetirizine, topical corticosteroids or antipruritic agents may be prescribed.

Subjects should be advised to contact the investigator immediately if there is any worsening of the acute systemic allergic reaction, in which case the subject will permanently discontinue the intake of trial medication and be withdrawn from the trial. Rechallenge is not allowed.

**Grade 3 (Generalized Urticaria, Angioedema With Medical Intervention Indicated,**

Symptomatic Mild Bronchospasm) and Grade 4 (Acute Anaphylaxis, Life-Threatening Bronchospasm, or Laryngeal Edema)

Subjects will permanently discontinue the intake of trial medication and be withdrawn from the trial. Rechallenge is not allowed.

Subjects will be treated as clinically appropriate. Subjects should be followed until resolution of the AE and standard management should be undertaken.

**AST AND ALT ELEVATION**

Grade 1 (≥ 1.25 to ≤ 2.5 x ULN), or Grade 2 (> 2.5 to ≤ 5.0 x ULN)

Subjects may continue the intake of trial medication.

Subjects should be followed until resolution (return to baseline) or stabilization of AST/ALT elevation.

Grade 3 (> 5.0 to ≤ 10.0 x ULN), or Grade 4 (> 10.0 x ULN)

Subjects will permanently discontinue the intake of trial medication and be withdrawn from the trial.

Subjects should be followed until resolution (return to baseline) or stabilization of AST/ALT elevation.

**CLINICAL HEPATITIS**

Subjects taking the trial medication should be monitored for the development of signs and symptoms of hepatitis which include fatigue, malaise, anorexia, nausea, dark urine and clay colored stools, bilirubinuria, jaundice, liver tenderness, or hepatomegaly, with or without initially abnormal serum transaminase levels.

Subjects with these signs and symptoms must seek medical attention immediately and have hepatic parameters assessed. Relevant markers of viral hepatitis should also be assessed. For safety reasons, if the hepatitis is deemed by the investigators to be possibly caused by study medications, additional drug level measurements may be performed to ensure that the medications have been cleared from the body.

Subjects with hepatitis will permanently discontinue the intake of trial medication and be withdrawn from the trial. Subjects need to be followed until resolution of the AE.

GASTROINTESTINAL NAUSEA (WITH OR WITHOUT VOMITING)

Although common, nausea following initiation of therapy with antiretroviral medication usually subsides or resolves during the first few weeks of treatment.

**Grade 1 (Transient [< 24 Hours] or Intermittent Nausea With no or Minimal Interference With Oral Intake)**

Subjects may continue the intake of trial medication and may be treated as needed with

antiemetics given orally or rectally.

**Grade 2 (Persistent Nausea Resulting in Decreased Oral Intake for 24 to 48 Hours)**

Subjects may be treated as needed with antiemetics given orally or rectally. If the nausea persists (>48 hours) despite antiemetics or increases in severity, subjects will permanently discontinue the intake of trial medication and be withdrawn from the trial.

**Grade 3 (Persistent Nausea Resulting in Minimal Oral Intake for > 48 Hours, or Aggressive Rehydration Indicated) and Grade 4 (Life-Threatening Consequences)** Subjects will permanently discontinue the intake of trial medication and be withdrawn from the trial. Rechallenge is not allowed.

**DIARRHOEA**

**Grade 1 (Transient or Intermittent Episodes of Unformed Stools, or Increase of ≤ 3 Stools Over Baseline per 24-Hour Period)**

Subjects may continue the intake of trial medication. Loperamide can be administered.

**Grade 2 (Persistent Episodes of Unformed to Watery Stools, or Increase of 4 to 6 Stools Over Baseline per 24-Hour Period)**

Subjects may continue the intake of trial medication. Loperamide can be administered.

**Grade 3 (Bloody Diarrhea, Increase of ≥ 7 Stools per 24-Hour Period, or i.v. Fluid Replacement Indicated), or Grade 4 (Life-Threatening Consequences)** will result in trial discontinuation. Subjects should be followed until resolution of the AE. Rechallenge is not allowed.

**11.3 REMOVAL OF SUBJECTS FROM THERAPY OR ASSESSMENT**

Subjects should be withdrawn from the trial if a **SAE** occurs. Subjects **must** also be withdrawn from the trial if:

1. they withdraw their consent;

2. the investigator considers it in the best interest of the subject that he or she is withdrawn;

3. they experience a grade 3 or 4 AE/toxicity;

4. they experience a grade 2 or higher rash;

5. they develop clinical hepatitis;

6. they become pregnant during study period.

1. they develop a renal complication considered related to the trial medication by the investigator.
2. they develop neutrophil count <600 mm3 will be grounds for treatment discontinuation.

The date and the reason for discontinuation must be noted in the source document. All subjects prematurely discontinuing from the trial must be seen, if consent is not withdrawn, for an early termination visit. If discontinuation from the trial was due to an AE, subjects will be followed until resolution or stabilization of AE. Haematological and chemistry tests will be performed on discontinuation and followed till resolution.

12.0 ADVERSE EVENTS

12.1 Importance of Adverse Event Reporting

Timely and complete reporting of safety information assists in identifying any untoward medical occurrence, thereby allowing: (1) protection of safety of study subjects; (2) a greater understanding of the overall safety profile of the co-administered products; (3) recognition of dose-related toxicity; (4) appropriate modification of study protocols; (5) improvements in study design or procedures; and (6) adherence to worldwide regulatory requirements.

12.2 Collection of Safety Information

An ***Adverse Event* *(AE)*** is defined as any new untoward medical occurrence or worsening of a pre-existing medical condition in a subject administered a medicinal product and which does not necessarily have a causal relationship with this treatment. An AE can therefore be any unfavorable and unintended sign (including an abnormal laboratory finding, for example), symptom, or disease temporally associated with the use of a medicinal (investigational or marketed) product, whether or not considered related to the medicinal (investigational or marketed) product.

Adverse events can be spontaneously reported or elicited during open-ended questioning, examination, or evaluation of a subject. (In order to prevent reporting bias, patients should not be questioned regarding the specific occurrence of one or more adverse events.)

Following the subject’s written consent to participate in the study, all serious AEs will be collected until completion of the last study-related procedure. The collection of non-serious AE information will begin at initiation of investigational product until completion of the last study-related procedure.

All identified AEs will be recorded and described on the CRF. If known, the diagnosis of the underlying illness or disorder should be recorded, rather than its individual symptoms. The following information should be captured for all AEs: date (and time) of onset and resolution, severity of the event (see appendix II), investigator’s opinion of the relationship to investigational product (see definitions), treatment/measures required for the AE, cause of the event (if known), and information regarding resolution/outcome.

The following categories and definitions of causal relationship to study drug will be used for all clinical trial AEs:

- Very Likely: There is a reasonable causal relationship between the study drug and the AE. The relationship in time is very suggestive. The event responds to withdrawal of study drug (dechallenge), and recurs with rechallenge when clinically feasible. It cannot be reasonably explained by an alternative explanation.
- Probable: There is a reasonable causal relationship between the study drug and the AE. The relationship in time is suggestive. The event responds to dechallenge. Rechallenge is not required. An alternative explanation is less likely.
- Possible: There is reasonable causal relationship between the study drug and the AE. The relationship in time is reasonable; therefore, the causal relationship cannot be excluded. Dechallenge information is lacking or unclear. An alternative explanation is inconclusive.
- Doubtful: An alternative explanation is more likely or the relationship in time suggests that a causal relationship is unlikely.
- Not Related: There is not a temporal relationship to study drug administration (too early, or late, or study drug not taken), or there is a reasonable causal relationship between another drug, concurrent disease, or circumstance and the AE.

An adverse event is considered associated with the use of the drug if the attribution is possible, probable, or very likely by the definitions listed above.

An unlisted (unexpected) adverse event, the nature or severity of which is not consistent with the applicable product information. For an investigational product, the expectedness of an adverse event will be determined by whether or not it is listed in the Investigator's Brochure. For a comparator product with a marketing authorization, the expectedness of an adverse event will be determined by whether or not it is listed in the Summary of Product Characteristics (SmPC).

12.3 Overdose

An overdose is defined as the accidental or intentional ingestion of any dose of a product that is considered both excessive and medically important. For reporting purposes, the investigator considers an overdose, regardless of adverse outcome, as an important medical event (see Serious Adverse Events).

12.4 AE Follow-up

AEs will be followed to resolution or stabilization, and reported as SAEs if they become serious. This also applies to subjects experiencing AEs that cause interruption or discontinuation of investigational product, or those experiencing AEs that are present at the end of their participation in the study; such subjects should receive post-treatment follow-up as appropriate. If an ongoing AE changes in its severity or in its perceived relationship to study drug, a new AE entry for the event should be completed.

12.5 Reporting of AE Information Following Study Completion

Collection of safety information following the end of investigational product administration is important in assisting in the identification of possible delayed toxicities or withdrawal effects. All SAEs must be collected which occur within 30 days of discontinuation of dosing or completion of the patient’s participation in the study if the last scheduled visit occurs at a later time. In addition, the investigator should notify the DSRB of any SAE which may occur after this time period which they believe to be related to the investigational product.

12.6 Handling of Serious Adverse Events (SAEs)

A ***serious AE*** is any untoward medical occurrence that at any dose:

- results in death,
- is life-threatening (defined as an event in which the subject or patient was at risk of death at the time of the event; it does not refer to an event which hypothetically might have caused death if it were more severe),
- requires inpatient hospitalization or causes prolongation of existing hospitalization,
- results in persistent or significant disability/incapacity,
- is a congenital anomaly/birth defect,
- results in the development of drug dependency or drug abuse is an important medical event [defined as a medical event(s) that may not be immediately life-threatening or result in death or hospitalization but, based upon appropriate medical and scientific judgment, may jeopardize the patient/subject or may require intervention (e.g., medical, surgical) to prevent one of the other serious outcomes listed in the definition above.]

The investigator assumes responsibility for appropriate reporting of Serious Adverse Events to the regulatory authorities. All serious adverse events occurring during clinical studies must be reported to the appropriate DSRB by investigational staff within 24 hours of their knowledge of the event.

All serious adverse events that have not resolved by the end of the study, or that have not resolved upon discontinuation of the subject’s participation in the study, must be followed until any of the following occurs:

- the event resolves
- the event stabilizes
- the event returns to baseline, if a baseline value is available
- the event can be attributed to agents other than the study drug or to factors unrelated to study conduct
- when it becomes unlikely that any additional information can be obtained (subject or health care practitioner refusal to provide additional information, lost to follow-up after demonstration of due diligence with follow-up efforts)

The cause of death of a subject in a clinical study, whether or not the event is expected or associated with the investigational agent, is considered a serious adverse event. Any event requiring hospitalization (or prolongation of hospitalization) that occurs during the course of a subject’s participation in a clinical study must be reported as a serious adverse event, except hospitalizations for:

- social reasons in absence of an adverse event
- surgery or procedure planned before entry into the study

Collection of complete information concerning SAEs is extremely important. If only limited information is initially available, follow-up reports are required. Also, follow-up information which becomes available as the SAE evolves, as well as supporting documentation (e.g., hospital discharge summaries and autopsy reports), will be collected subsequently, if not available at the time of the initial report, and immediately sent using the same procedure as the initial SAE report.

12.7 Laboratory Test Abnormalities

All clinically significant laboratory abnormalities will be captured on the AE pages of the CRF.

12.8 Other Safety Considerations

Any clinically significant changes noted during interim or final physical examinations and any other potential safety assessments, whether or not these procedures are required by the protocol, should also be recorded on the AE page of the CRF

12.9 Safety Reporting Contact information

**SAE TELEPHONE CONTACT:**

Lawrence Lee, MBBS, MRCP(UK), PhD.

Phone: 6779-5555

mdcllsu@nus.edu.sg

**13.0 HUMAN SUBJECTS AND STUDY CONDUCT**

**13.1 Study Conduct**

This study will be conducted in accordance with the ethical principles that have their origin in the current Declaration of Helsinki and will be consistent with International Conference on Harmonization Good Clinical Practice (ICH GCP) and applicable regulatory requirements.

The study will be conducted in compliance with the protocol. The protocol and any Amendments and the subject informed consent will receive Institutional Review Board (IRB)/Independent Ethics Committee (IEC) approval/favorable opinion prior to initiation of the study.

Freely given written informed consent will be obtained from every subject prior to clinical trial participation, including informed consent for any screening procedures conducted to establish subject eligibility for the trial. The rights, safety and well-being of the trial subjects are the most important considerations.

**13.2 Institutional Review Board and Informed Consent**

This protocol and the informed consent document and any subsequent modifications will be reviewed and approved by the IRB or ethics committee responsible for oversight of the study. A signed consent form will be obtained from the subject. The subject's consent must be obtained to ensure that he or she is able to understand the nature, significance, and risks associated with the study. The consent form will describe the purpose of the study, the procedures to be followed, and the risks and benefits of participation. A copy of the consent form will be given to the subject, parent, or legal guardian, and this fact will be documented in the subject’s record.

**13.3 Confidentiality**

All laboratory specimens, evaluation forms, reports, and other records that leave the site will be identified by coded number only to maintain subject confidentiality. All records will be kept in a locked file cabinet. Clinical information will not be released without written permission of the subject, except as necessary for monitoring by the IRB or the HSA.

A quality assurance audit of this trial may be conducted by an outside contractor. The quality assurance auditor will have access to all medical records, the investigator’s trial related files and correspondence, and the informed consent documentation that is relevant to this trial.

**13.4 Pregnancy**

Sexually active women of childbearing potential must use an effective method of birth control as described in the Inclusion Criteria of the study. Women of childbearing potential (WOCBP) must practice birth control during the course of the study and for 30 days post the last dose of study medication. Male study volunteers will be required to use a barrier method during the study and for at least 3 months after completion of the study.

All WOCBP MUST have a negative pregnancy test within 24 hours prior to receiving investigational product. If the pregnancy test is positive, the subject must not receive investigational product and must not be enrolled in the study.

Pregnancy testing must also be performed throughout the study as specified in Section 6.0 and the results of all pregnancy tests (positive or negative) recorded on the case report form.

In addition, all WOCBP should be instructed to contact the Investigator immediately if they suspect they might be pregnant (e.g., missed or late menstrual period) at any time during study participation.

If following initiation of study treatment, it is subsequently discovered that a trial subject is pregnant or may have been pregnant at the time of investigational product administration the investigational product will be permanently discontinued. All pregnancies should be reported to the Antiretroviral Pregnancy Registry (APR) at the time the pregnancy is confirmed. Pregnancy outcome information should subsequently be reported to the APR.

Protocol-required procedures for study discontinuation and follow-up must be performed on the subject.

**References**

1. Lim, Y.W., et al., *Pharmacokinetics and pharmacodynamics of docetaxel with or without ketoconazole modulation in chemonaive breast cancer patients.* Ann Oncol.

2. Yong, W.P., et al., *A phase I study of docetaxel with ketoconazole modulation in patients with advanced cancers.* Cancer Chemother Pharmacol, 2008. 62(2): p. 243-51.

3. Hor, S.Y., et al., *PXR, CAR and HNF4alpha genotypes and their association with pharmacokinetics and pharmacodynamics of docetaxel and doxorubicin in Asian patients.* Pharmacogenomics J, 2008. 8(2): p. 139-46.

4. Thummel, K.E., et al., *Use of midazolam as a human cytochrome P450 3A probe: I. In vitro-in vivo correlations in liver transplant patients.* J Pharmacol Exp Ther, 1994. 271(1): p. 549-56.

5. Kassahun, K., et al., *Metabolism and disposition in humans of raltegravir (MK-0518), an anti-AIDS drug targeting the human immunodeficiency virus 1 integrase enzyme.* Drug Metab Dispos, 2007. 35(9): p. 1657-63.

6. Iwamoto, M., et al., *Lack of a pharmacokinetic effect of raltegravir on midazolam: in vitro/in vivo correlation.* J Clin Pharmacol, 2008. 48(2): p. 209-14.

7. Greenblatt, D.J., et al., *Inhibition of oral midazolam clearance by boosting doses of ritonavir, and by 4,4-dimethyl-benziso-(2H)-selenazine (ALT-2074), an experimental catalytic mimic of glutathione oxidase.* Br J Clin Pharmacol, 2009. 68(6): p. 920-7.

8. Stoch, S.A., et al., *Effect of different durations of ketoconazole dosing on the single-dose pharmacokinetics of midazolam: shortening the paradigm.* J Clin Pharmacol, 2009. 49(4): p. 398-406.

9. Oostendorp, R.L., et al., *Coadministration of ritonavir strongly enhances the apparent oral bioavailability of docetaxel in patients with solid tumors.* Clin Cancer Res, 2009. 15(12): p. 4228-33.

10. Yong, W.P., et al., *Effects of ketoconazole on glucuronidation by UDP-glucuronosyltransferase enzymes.* Clin Cancer Res, 2005. 11(18): p. 6699-704.

11. Wenning, L.A., et al., *Effect of Rifampin, a Potent Inducer of Drug Metabolizing Enzymes, on the Pharmacokinetics of Raltegravir.* Antimicrob Agents Chemother, 2009.

12. Abbott, I., *Norvir (ritonavir) tablets. FDA approved label.* 2009.

13. Merck &Co Inc., *Isentress (raltegravir) tablets. FDA approved label.* 2007.

**Appendix I**

**Procedure for Collection, Storage and Shipping of Pharmacokinetic Samples**

**Blood Collection**

3.0 mL samples of blood will be collected into a Lithium Heparin Vacutainer® tube at the exact times specified in the protocol. The whole blood sample should be placed at room temperature. A too small or too large needle used to collect blood samples may cause haemolysis of the blood samples. Care should be taken to avoid haemolysis.

8.0 mL samples of blood will be collected into BD CPT cell separation tubes at 3 hours post dose. Cells will then be washed 3 times, then counted and sized using a handheld Millipore device. The washed counted cells will be stored in methanol at -80C.

Preprinted labels for blood tubes will be supplied by the site. They will include protocol number, study day, nominal sample time, sample code and subject number. Labels should be fixed lengthwise to room temperature tubes. The actual date, and nominal and clock sampling times should be recorded on the CRFs. Any important information concerning the sample or its collection (i.e., missed or late sample, haemolysis, etc) must be recorded in the ‘Remarks’ section of this CRF page.

All entries on the CRFs and on spare or blank tube labels should be in indelible ink.

**Sample Storage**

Blood samples immediately will be stored in an upright position at room temperature.

**Sample Dispatch and Analysis**

Whole blood samples will be transported to Prof Yong Eu Leong’s lab at 04-18 MD11, Clinical Research Centre, Attn: Ms Soon Gaik Hong.

**APPENDIX II : DAIDS AE Grading Table**

The Division of AIDS Table for Grading the Severity of Adult and Pediatric AEs (“DAIDS AE grading table”) is a descriptive terminology to be utilized for AE reporting in this trial. A grading (severity) scale is provided for each AE term.

**General Instructions**

*Estimating Severity Grade*

If the need arises to grade a clinical AE that is not identified in the DAIDS AE grading table, use the category “Estimating Severity Grade” located at the top of the table on the following page.

*Grading Adult and Pediatric AEs*

The DAIDS AE grading table includes parameters for grading both Adult and Pediatric AEs. When a single set of parameters is not appropriate for grading specific types of AEs for both Adult and Pediatric populations, separate sets of parameters for Adult and/or Pediatric populations (with specified respective age ranges) are provided. If there is no distinction in the table between Adult and Pediatric values for a type of AE, then the single set of parameters listed is to be used for grading the severity of both Adult and Pediatric events of that type.

*Determining Severity Grade*

***If the severity of an AE could fall under either one of 2 grades (e.g., the severity of an AE could be either grade 2 or grade 3), select the higher of the 2 grades for the AE.***

Definitions

|  | | | Basic self-care functions | | | | Adult: activities such as bathing, dressing, toileting, transfer/movement, continence, and feeding. | | | | | | | | | |  | | |
| --- | --- | --- | --- | --- | --- | --- | --- | --- | --- | --- | --- | --- | --- | --- | --- | --- | --- | --- | --- |
|  | | |  | | | |  | | | | | | | | | |  | | |
|  | | | Usual social & functional activities | | | | Adult: adaptive tasks and desirable activities, such as going to work, shopping, cooking, use of transportation, pursuing a hobby, etc. | | | | | | | | | |  | | |
|  | | |  | | | |  | | | | | | | | | |  | | |
|  | | | Medical intervention | | | | Use of pharmacologic or biologic agent(s) for treatment of an AE. | | | | | | | | | |  | | |
|  | | | Operative intervention | | | | Surgical OR other invasive mechanical procedures. | | | | | | | | | |  | | |
|  | **CLINICAL** | | | | | | | | | | | | | | | | | | |
|  | **PARAMETER** | | | | **GRADE 1**  **MILD** | | | | **GRADE 2**  **MODERATE** | | | **GRADE 3**  **SEVERE** | | | **GRADE 4**  **POTENTIALLY** LIFE-THREATENING | | | | |
|  | **ESTIMATING SEVERITY GRADE** | | | | | | | | | | | | | | | | | | |
|  | Clinical adverse event NOT identified elsewhere in this DAIDS AE grading table | | | | Symptoms causing no or minimal interference with usual social & functional activities | | | | Symptoms causing greater than minimal interference with usual social & functional activities | | | Symptoms causing inability to perform usual social & functional activities | | | Symptoms causing inability to perform basic self-care functions OR Medical or operative intervention indicated to prevent permanent impairment, persistent disability, or death | | | | |
|  | **SYSTEMIC** | | | | | | | | | | | | | | | | | | |
|  | Acute systemic allergic reaction | | | | Localized urticaria (wheals) with no medical intervention indicated | | | | Localized urticaria with medical intervention indicated OR Mild angioedema with no medical intervention indicated | | | Generalized urticaria OR Angioedema with medical intervention indicated OR Symptomatic mild bronchospasm | | | Acute anaphylaxis OR Life-threatening bronchospasm OR Laryngeal edema | | | | |
|  | Chills | | | | Symptoms causing no or minimal interference with usual social & functional activities | | | | Symptoms causing greater than minimal interference with usual social & functional activities | | | Symptoms causing inability to perform usual social & functional activities | | | NA | | | | |
|  | Fatigue  Malaise | | | | Symptoms causing no or minimal interference with usual social & functional activities | | | | Symptoms causing greater than minimal interference with usual social & functional activities | | | Symptoms causing inability to perform usual social & functional activities | | | Incapacitating fatigue/ malaise symptoms causing inability to perform basic self-care functions | | | | |
|  | Fever (nonaxillary) | | | | 37.7°C – 38.6°C | | | | 38.7°C – 39.3°C | | | 39.4°C – 40.5°C | | | > 40.5°C | | | | |
|  | Pain (indicate body site). DO NOT use for pain due to injection (See Injection site reactions: Injection site pain). See also Headache, Arthralgia, and Myalgia | | | | Pain causing no or minimal interference with usual social & functional activities | | | | Pain causing greater than minimal interference with usual social & functional activities | | | Pain causing inability to perform usual social & functional activities | | | Disabling pain causing inability to perform basic self-care functions OR Hospitalization (other than emergency room visit) indicated | | | | |
|  | Unintentional weight loss | | | | NA | | | | 5% – 9% loss in body weight from baseline | | | 10% – 19% loss in body weight from baseline | | | ≥ 20% loss in body weight from baseline OR Aggressive intervention indicated [e.g., tube feeding or total parenteral nutrition (TPN)] | | | | |
| CLINICAL | | | | | | | | | | | | | | | | | | |  |
| **PARAMETER** | | | | **GRADE 1**  **MILD** | | | | **GRADE 2**  **MODERATE** | | | **GRADE 3**  **SEVERE** | | | **GRADE 4**  **POTENTIALLY LIFE-THREATENING** | | | | |  |
| **INFECTION** | | | | | | | | | | | | | | | | | | |  |
| Infection (any other than HIV infection) | | | | Localized, no systemic antimicrobial treatment indicated AND Symptoms causing no or minimal interference with usual social & functional activities | | | | Systemic antimicrobial treatment indicated OR Symptoms causing greater than minimal interference with usual social & functional activities | | | Systemic antimicrobial treatment indicated AND Symptoms causing inability to perform usual social & functional activities OR Operative intervention (other than simple incision and drainage) indicated | | | Life-threatening consequences (e.g., septic shock) | | | | |  |
| **INJECTION SITE REACTIONS** | | | | | | | | | | | | | | | | | | |  |
| Injection site pain (pain without touching)  Or  Tenderness (pain when area is touched) | | | | Pain/tenderness causing no or minimal limitation of use of limb | | | | Pain/tenderness limiting use of limb OR Pain/tenderness causing greater than minimal interference with usual social & functional activities | | | Pain/tenderness causing inability to perform usual social & functional activities | | | Pain/tenderness causing inability to perform basic self-care function OR Hospitalization (other than emergency room visit) indicated for management of pain/tenderness | | | | |  |
| Injection site reaction (localized) | | | | | | | | | | | | | | | | | | |  |
|  | | **Adult > 15 years** | | Erythema OR Induration  of 5 x 5 cm – 9 x 9 cm (or 25 cm² – 81cm²) | | | | Erythema OR Induration OR Edema > 9 cm any diameter  (or > 81 cm²) | | | Ulceration OR Secondary infection OR Phlebitis OR Sterile abscess OR Drainage | | | Necrosis (involving dermis and deeper tissue) | | | | |  |
|  | |  | |  | | | |  | | |  | | |  | | | | |  |
| Pruritis associated with injection  See also Skin: Pruritis (itching - no skin lesions) | | | | Itching localized to injection site AND Relieved spontaneously or with < 48 hours treatment | | | | Itching beyond the injection site but not generalized OR Itching localized to injection site requiring ≥ 48 hours treatment | | | Generalized itching causing inability to perform usual social & functional activities | | | NA | | | | |  |
| **CLINICAL** | | | | | | | | | | | | | | | | | |  | |
| **PARAMETER** | | | | | | **GRADE 1**  **MILD** | | | | **GRADE 2**  **MODERATE** | | | **GRADE 3**  **SEVERE** | | | **GRADE 4**  POTENTIALLY LIFE-THREATENING | |  | |
| **SKIN – DERMATOLOGICAL** | | | | | | | | | | | | | | | | | |  | |
| Alopecia | | | | | | Thinning detectable by study participant (or by caregiver for young children and disabled adults) | | | | Thinning or patchy hair loss detectable by health care provider | | | Complete hair loss | | | NA | |  | |
| Cutaneous reaction/rash | | | | | | Localized macular rash | | | | Diffuse macular, maculopapular, or morbilliform rash OR target lesions | | | Diffuse macular, maculopapular, or morbilliform rash with vesicles or limited number of bullae OR superficial ulcerations of mucous membrane limited to 1 site^a^ OR diffuse maculopapular rash with at least 1 of the following^a^: Elevation of AST and/or ALT > 2 x baseline but at least > 5 x ULN^a^; Fever (> 38°C or 100°F) ^a^; Eosinophils > 1000/mm^3a^; Serum sickness-like reaction^a^ | | | Extensive or generalized bullous lesions OR Stevens-Johnson syndrome (SJS) OR ulceration of mucous membrane involving 2 or more distinct mucosal sites OR toxic epidermal necrolysis (TEN) | |  | |
| Hyperpigmentation | | | | | | Slight or localized | | | | Marked or generalized | | | NA | | | NA | |  | |
| Hypopigmentation | | | | | | Slight or localized | | | | Marked or generalized | | | NA | | | NA | |  | |
| Pruritis (itching – no skin lesions)  (See also Injection site reactions: Pruritis associated with injection) | | | | | | Itching causing no or minimal interference with usual social & functional activities | | | | Itching causing greater than minimal interference with usual social & functional activities | | | Itching causing inability to perform usual social & functional activities | | | NA | |  | |
| **CARDIOVASCULAR** | | | | | | | | | | | | | | | | | |  | |
| Cardiac arrhythmia (general) (By ECG or physical exam) | | | | | | Asymptomatic AND No intervention indicated | | | | Asymptomatic AND Nonurgent medical intervention indicated | | | Symptomatic, non-life threatening AND Nonurgent medical intervention indicated | | | Life-threatening arrhythmia OR Urgent intervention indicated | |  | |
| Cardiac ischemia/ infarction | | | | | | NA | | | | NA | | | Symptomatic ischemia (stable angina) OR Testing consistent with ischemia | | | Unstable angina OR Acute myocardial Infarction | |  | |
| Haemorrhage (significant acute blood loss) | | | | | | NA | | | | Symptomatic AND No transfusion indicated | | | Symptomatic AND Transfusion of ≤ 2 units packed RBCs (for children ≤ 10 cc/kg) indicated | | | Life-threatening hypotension OR Transfusion of > 2 units packed RBCs (for children > 10 cc/kg) indicated | |  | |

^a^ Revised by Tibotec Pharmaceuticals Ltd.

| **CLINICAL** | | | | | |
| --- | --- | --- | --- | --- | --- |
| **PARAMETER** | | **GRADE 1**  **MILD** | **GRADE 2**  **MODERATE** | **GRADE 3**  **SEVERE** | **GRADE 4**  POTENTIALLY LIFE-THREATENING |
| Hypertension | | | | | |
|  | **Adult > 17 years**  (with repeat testing at same visit) | > 140 – 159 mmHg systolic  OR  > 90 – 99 mmHg diastolic | > 160 – 179 mmHg systolic  OR  > 100 – 109 mmHg diastolic | > 180 mmHg systolic  OR  > 110 mmHg diastolic | Life-threatening consequences (e.g., malignant hypertension) OR Hospitalization indicated (other than emergency room visit) |
|  |  |  |  |  |  |
| Hypotension | | NA | Symptomatic, corrected with oral fluid replacement | Symptomatic, i.v. fluids indicated | Shock requiring use of vasopressors or mechanical assistance to maintain blood pressure |
| Pericardial effusion | | Asymptomatic, small effusion requiring no intervention | Asymptomatic, moderate or larger effusion requiring no intervention | Effusion with non-life threatening physiologic consequences OR Effusion with nonurgent intervention indicated | Life-threatening consequences (e.g., tamponade) OR Urgent intervention indicated |
| Prolonged PR interval | | | | | |
|  | **Adult > 16 years** | 1st degree AV block* | Second degree Type Mobitz I or Wenckebach* | Type II 2nd degree AV block OR Ventricular pause | Complete AV block |
|  |  |  |  |  |  |
| Thrombosis/embolism | | NA | Deep vein thrombosis AND No intervention indicated (e.g., anticoagulation, lysis filter, invasive procedure) | Deep vein thrombosis AND Intervention indicated (e.g., anticoagulation, lysis filter, invasive procedure) | Embolic event (e.g., pulmonary embolism, life-threatening thrombus) |
| Vasovagal episode (associated with a procedure of any kind) | | Present without loss of consciousness | Present with transient loss of consciousness | NA | NA |
| Ventricular dysfunction (congestive heart failure) | | NA | Asymptomatic diagnostic finding AND intervention indicated | New onset with symptoms OR Worsening symptomatic congestive heart failure | Life-threatening congestive heart failure |

* Adapted by Tibotec Pharmaceuticals Ltd.

| **CLINICAL** | | | | | |
| --- | --- | --- | --- | --- | --- |
| **PARAMETER** | | **GRADE 1**  **MILD** | **GRADE 2**  **MODERATE** | **GRADE 3**  **SEVERE** | **GRADE4 – Potentially**  **Life Threatening** |
| **GASTROINTESTINAL** | | | | | |
| Anorexia | | Loss of appetite without decreased oral intake | Loss of appetite associated with decreased oral intake without significant weight loss | Loss of appetite associated with significant weight loss | Life-threatening consequences OR Aggressive intervention indicated (e.g., tube feeding or total parenteral nutrition [TPN]) |
| Ascites | | Asymptomatic | Symptomatic AND Intervention indicated (e.g., diuretics or therapeutic paracentesis) | Symptomatic despite intervention | Life-threatening consequences |
| Cholecystitis | | NA | Symptomatic AND Medical intervention indicated | Radiologic, endoscopic, or operative intervention indicated | Life-threatening consequences (e.g., sepsis or perforation) |
| Constipation | | NA | Persistent constipation requiring regular use of dietary modifications, laxatives, or enemas | Obstipation with manual evacuation indicated | Life-threatening consequences (e.g., obstruction) |
| Diarrhea | | | | | |
|  | **Adult and Pediatric ≥ 1 year** | Transient or intermittent episodes of unformed stools OR Increase of ≤ 3 stools over baseline per  24-hour period | Persistent episodes of unformed to watery stools OR Increase of  4 – 6 stools over baseline per 24-hour period | Bloody diarrhea OR Increase of ≥ 7 stools per 24-hour period OR i.v. fluid replacement indicated | Life-threatening consequences (e.g., hypotensive shock) |
|  |  |  |  |  |  |
| Dysphagia-Odynophagia | | Symptomatic but able to eat usual diet | Symptoms causing altered dietary intake without medical intervention indicated | Symptoms causing severely altered dietary intake with medical intervention indicated | Life-threatening reduction in oral intake |
| Mucositis/stomatitis (clinical exam) Indicate site (e.g., larynx, oral)  See Genitourinary for Vulvovaginitis. See also Dysphagia- Odynophagia and Proctitis | | Erythema of the Mucosa | Patchy pseudomembranes or ulcerations | Confluent pseudomembranes or ulcerations OR Mucosal bleeding with minor trauma | Tissue necrosis OR Diffuse spontaneous mucosal bleeding OR Life-threatening consequences (e.g., aspiration, choking) |
| Nausea | | Transient (< 24 hours) or intermittent nausea with no or minimal interference with oral intake | Persistent nausea resulting in decreased oral intake for  24 – 48 hours | Persistent nausea resulting in minimal oral intake for  > 48 hours OR Aggressive rehydration indicated (e.g., i.v. fluids) | Life-threatening consequences (e.g., hypotensive shock) |

| **CLINICAL** | | | | | | | | |
| --- | --- | --- | --- | --- | --- | --- | --- | --- |
| **PARAMETER** | **GRADE 1**  **MILD** | | **GRADE 2**  **MODERATE** | | **GRADE 3**  **SEVERE** | | **GRADE 4**  **POTENTIALLY LIFE-THREATENING** | |
| Pancreatitis | NA | | Symptomatic AND Hospitalization not indicated (other than emergency room visit) | | Symptomatic AND Hospitalization indicated (other than emergency room visit) | | Life-threatening consequences (e.g., circulatory failure, haemorrhage, sepsis) | |
| Proctitis (functional- symptomatic)  Also see Mucositis/stomatitis for clinical exam | Rectal discomfort AND No intervention Indicated | | Symptoms causing greater than minimal interference with usual social & functional activities OR Medical intervention indicated | | Symptoms causing inability to perform usual social & functional activities OR Operative intervention indicated | | Life-threatening consequences (e.g., perforation) | |
| Vomiting | Transient or intermittent vomiting with no or minimal interference with oral intake | | Frequent episodes of vomiting with no or mild dehydration | | Persistent vomiting resulting in orthostatic hypotension OR Aggressive rehydration indicated (e.g., i.v. fluids) | | Life-threatening consequences (e.g., hypotensive shock) | |
| **NEUROLOGIC** | | | | | | | | |
| Alteration in personality-behavior or in mood (e.g., agitation, anxiety, depression, mania, psychosis) | | Alteration causing no or minimal interference with usual social & functional activities | | Alteration causing greater than minimal interference with usual social & functional activities | | Alteration causing inability to perform usual social & functional activities | | Behavior potentially harmful to self or others (e.g., suicidal and homicidal ideation or attempt, acute psychosis) OR Causing inability to perform basic self-care functions |
| Altered Mental Status  For Dementia, see Cognitive and behavioral/attentional disturbance (including dementia and attention deficit disorder) | | Changes causing no or minimal interference with usual social & functional activities | | Mild lethargy or somnolence causing greater than minimal interference with usual social & functional activities | | Confusion, memory impairment, lethargy, or somnolence causing inability to perform usual social & functional activities | | Delirium OR obtundation, OR coma |
| Ataxia | | Asymptomatic ataxia detectable on exam OR Minimal ataxia causing no or minimal interference with usual social & functional activities | | Symptomatic ataxia causing greater than minimal interference with usual social & functional activities | | Symptomatic ataxia causing inability to perform usual social & functional activities | | Disabling ataxia causing inability to perform basic self-care functions |
| Cognitive and behavioral/attentional disturbance (including dementia and attention deficit disorder) | | Disability causing no or minimal interference with usual social & functional activities OR Specialized resources not indicated | | Disability causing greater than minimal interference with usual social & functional activities OR Specialized resources on part-time basis indicated | | Disability causing inability to perform usual social & functional activities OR Specialized resources on a full-time basis indicated | | Disability causing inability to perform basic self-care functions OR Institutionalization Indicated |
| CNS ischemia (acute) | | NA | | NA | | Transient ischemic Attack | | Cerebral vascular accident (CVA, stroke) with neurological deficit |

| **CLINICAL** | | | | |
| --- | --- | --- | --- | --- |
| **PARAMETER** | **GRADE 1**  **MILD** | **GRADE 2**  **MODERATE** | **GRADE 3**  **SEVERE** | **GRADE 4**  Potentially Life Threatenting |
|  |  |  |  |  |
| Headache | Symptoms causing no or minimal interference with usual social & functional activities | Symptoms causing greater than minimal interference with usual social & functional activities | Symptoms causing inability to perform usual social & functional activities | Symptoms causing inability to perform basic self-care functions OR Hospitalization indicated (other than emergency room visit) OR Headache with significant impairment of alertness or other neurologic function |
| Insomnia | NA | Difficulty sleeping causing greater than minimal interference with usual social & functional activities | Difficulty sleeping causing inability to perform usual social & functional activities | Disabling insomnia causing inability to perform basic self-care functions |
| Neuromuscular weakness (including myopathy & neuropathy) | Asymptomatic with decreased strength on exam OR Minimal muscle weakness causing no or minimal interference with usual social & functional activities | Muscle weakness causing greater than minimal interference with usual social & functional activities | Muscle weakness causing inability to perform usual social & functional activities | Disabling muscle weakness causing inability to perform basic self-care functions OR Respiratory muscle weakness impairing ventilation |
| Neurosensory alteration (including paresthesia and painful neuropathy) | Asymptomatic with sensory alteration on exam or minimal paresthesia causing no or minimal interference with usual social & functional activities | Sensory alteration or paresthesia causing greater than minimal interference with usual social & functional activities | Sensory alteration or paresthesia causing inability to perform usual social & functional activities | Disabling sensory alteration or paresthesia causing inability to perform basic self-care functions |
| Seizure: (new onset) **Adult ≥ 18 years**  See also Seizure: (known pre-existing seizure disorder) | NA | 1 seizure | 2 **–** 4 seizures | Seizures of any kind which are prolonged, repetitive (e.g., status epilepticus), or difficult to control (e.g., refractory epilepsy) |

| **CLINICAL** | | | | | |
| --- | --- | --- | --- | --- | --- |
| **PARAMETER** | | **GRADE 1**  **MILD** | **GRADE 2**  **MODERATE** | **GRADE 3**  **SEVERE** | **GRADE 4**  **POTENTIALLY LIFE-THREATENING** |
| Seizure: (known pre‑existing seizure disorder)  **Adult ≥ 18 years**  For worsening of existing epilepsy the grades should be based on an increase from previous level of control to any of these levels. | | NA | Increased frequency of pre-existing seizures (nonrepetitive) without change in seizure character OR Infrequent breakthrough seizures while on stable medication in a previously controlled seizure disorder | Change in seizure character from baseline either in duration or quality (e.g., severity or focality) | Seizures of any kind which are prolonged, repetitive (e.g., status epilepticus), or difficult to control (e.g., refractory epilepsy) |
|  | |  |  |  |  |
| Syncope (not associated with a procedure) | | NA | Present | NA | NA |
| Vertigo | | Vertigo causing no or minimal interference with usual social & functional activities | Vertigo causing greater than minimal interference with usual social & functional activities | Vertigo causing inability to perform usual social & functional activities | Disabling vertigo causing inability to perform basic self-care functions |
| **RESPIRATORY** | | | | | |
| Bronchospasm (acute) | | FEV1 or peak flow reduced to 70% **–** 80% | FEV1 or peak flow 50% **–** 69% | FEV1 or peak flow  25% **–** 49% | Cyanosis OR FEV1 or peak flow < 25% OR Intubation |
| Dyspnea or respiratory distress | | | | | |
|  | **Adult ≥ 14 years** | Dyspnea on exertion with no or minimal interference with usual social & functional activities | Dyspnea on exertion causing greater than minimal interference with usual social & functional activities | Dyspnea at rest causing inability to perform usual social & functional activities | Respiratory failure with ventilatory support indicated |
|  |  |  |  |  |  |
| **MUSCULOSKELETAL** | | | | | |
| Arthralgia  See also Arthritis | | Joint pain causing no or minimal interference with usual social & functional activities | Joint pain causing greater than minimal interference with usual social & functional activities | Joint pain causing inability to perform usual social & functional activities | Disabling joint pain causing inability to perform basic self-care functions |
| Arthritis  See also Arthralgia | | Stiffness or joint swelling causing no or minimal interference with usual social & functional activities | Stiffness or joint swelling causing greater than minimal interference with usual social & functional activities | Stiffness or joint swelling causing inability to perform usual social & functional activities | Disabling joint stiffness or swelling causing inability to perform basic self-care functions |

| **CLINICAL** | | | | | |
| --- | --- | --- | --- | --- | --- |
| **PARAMETER** | | **GRADE 1**  **MILD** | **GRADE 2**  **MODERATE** | **GRADE 3**  **SEVERE** | **GRADE 4**  **Potentially Life Threatening** |
| Bone Mineral Loss | | | | | |
|  | **Adult ≥ 21 years** | BMD t-score  -2.5 to -1.0 | BMD t-score < -2.5 | Pathological fracture (including loss of vertebral height) | Pathologic fracture causing life-threatening consequences |
|  |  |  |  |  |  |
| Myalgia  (noninjection site) | | Muscle pain causing no or minimal interference with usual social & functional activities | Muscle pain causing greater than minimal interference with usual social & functional activities | Muscle pain causing inability to perform usual social & functional activities | Disabling muscle pain causing inability to perform basic self-care functions |
| Osteonecrosis | | NA | Asymptomatic with radiographic findings AND No operative intervention indicated | Symptomatic bone pain with radiographic findings OR Operative intervention indicated | Disabling bone pain with radiographic findings causing inability to perform basic self-care functions |
| **GENITOURINARY** | | | | | |
| Cervicitis (symptoms)  (For use in studies evaluating topical study agents)  For other cervicitis see Infection: Infection (any other than HIV infection) | | Symptoms causing no or minimal interference with usual social & functional activities | Symptoms causing greater than minimal interference with usual social & functional activities | Symptoms causing inability to perform usual social & functional activities | Symptoms causing inability to perform basic self-care functions |
| Cervicitis (clinical exam) (For use in studies evaluating topical study agents)  For other cervicitis, see Infection: Infection (any other than HIV infection) | | Minimal cervical abnormalities on examination (erythema, mucopurulent discharge, or friability) OR Epithelial disruption < 25% of total surface | Moderate cervical abnormalities on examination (erythema, mucopurulent discharge, or friability) OR Epithelial disruption of  25% – 49% total surface | Severe cervical abnormalities on examination (erythema, mucopurulent discharge, or friability) OR Epithelial disruption 50% – 75% total surface | Epithelial disruption  > 75% total surface |
| Intermenstrual bleeding (IMB) | | Spotting observed by participant OR Minimal blood observed during clinical or colposcopic examination | Inter-menstrual bleeding not greater in duration or amount than usual menstrual cycle | Inter-menstrual bleeding greater in duration or amount than usual menstrual cycle | Haemorrhage with lifethreatening hypotension OR Operative intervention indicated |
| Urinary tract  obstruction (e.g., stone) | | NA | Signs or symptoms of urinary tract obstruction without hydronephrosis or renal dysfunction | Signs or symptoms of urinary tract obstruction with hydronephrosis or renal dysfunction | Obstruction causing lifethreatening Consequences |

| **CLINICAL** | | | | |
| --- | --- | --- | --- | --- |
| **PARAMETER** | **GRADE 1**  **MILD** | **GRADE 2**  **MODERATE** | **GRADE 3**  **SEVERE** | **GRADE 4**  **POTENTIALLY LIFE-THREATENING** |
| Vulvovaginitis (symptoms)  (Use in studies evaluating topical study agents)  For other vulvovaginitis see Infection: Infection (any other than HIV infection) | Symptoms causing no or minimal interference with usual social & functional activities | Symptoms causing greater than minimal interference with usual social & functional activities | Symptoms causing inability to perform usual social & functional activities | Symptoms causing inability to perform basic self-care functions |
| Vulvovaginitis (clinical exam)  (Use in studies evaluating topical study agents)  For other vulvovaginitis see Infection: Infection (any other than HIV infection) | Minimal vaginal abnormalities on examination OR Epithelial disruption  < 25% of total surface | Moderate vaginal abnormalities on examination OR Epithelial disruption of 25% *–* 49% total surface | Severe vaginal abnormalities on examination OR Epithelial disruption 50% *–* 75% total surface | Vaginal perforation OR Epithelial disruption > 75% total surface |
| **OCULAR/VISUAL** | | | | |
| Uveitis | Asymptomatic but detectable on exam | Symptomatic anterior uveitis OR Medical intervention indicated | Posterior or pan-uveitis OR Operative intervention indicated | Disabling visual loss in affected eye(s) |
| Visual changes (from baseline) | Visual changes causing no or minimal interference with usual social & functional activities | Visual changes causing greater than minimal interference with usual social & functional activities | Visual changes causing inability to perform usual social & functional activities | Disabling visual loss in affected eye(s) |
| **ENDOCRINE/METABOLIC** | | | | |
| Abnormal fat accumulation (e.g., back of neck, breasts, abdomen) | Detectable by study participant (or by caregiver for young children and disabled adults) | Detectable on physical exam by health care provider | Disfiguring OR Obvious changes on casual visual inspection | NA |
| Diabetes mellitus | NA | New onset without need to initiate medication OR Modification of current medications to regain glucose control | New onset with initiation of medication indicated OR Diabetes uncontrolled despite treatment modification | Life-threatening consequences (e.g., ketoacidosis, hyperosmolar nonketotic coma) |
| Gynecomastia | Detectable by study participant or caregiver (for young children and disabled adults) | Detectable on physical exam by health care provider | Disfiguring OR Obvious on casual visual inspection | NA |
| Hyperthyroidism | Asymptomatic | Symptomatic causing greater than minimal interference with usual social & functional activities OR Thyroid suppression therapy indicated | Symptoms causing inability to perform usual social & functional activities OR Uncontrolled despite treatment modification | Life-threatening consequences (e.g., thyroid storm) |

| **CLINICAL** | | | | |
| --- | --- | --- | --- | --- |
| **PARAMETER** | **GRADE 1**  **MILD** | **GRADE 2**  **MODERATE** | **GRADE 3**  **SEVERE** | **GRADE 4**  **POTENTIALLY LIFE-THREATENING** |
| Hypothyroidism | Asymptomatic | Symptomatic causing greater than minimal interference with usual social & functional activities OR Thyroid replacement therapy indicated | Symptoms causing inability to perform usual social & functional activities OR Uncontrolled despite treatment modification | Life-threatening consequences (e.g., myxedema coma) |
| Lipoatrophy (e.g., fat loss from the face, extremities, buttocks) | Detectable by study participant (or by caregiver for young children and disabled adults) | Detectable on physical exam by health care provider | Disfiguring OR Obvious on casual visual inspection | NA |

| **LABORATORY** | | | | | | | | | |
| --- | --- | --- | --- | --- | --- | --- | --- | --- | --- |
| **PARAMETER** | | | **GRADE 1**  **MILD** | | **GRADE 2**  **MODERATE** | | **GRADE 3**  **SEVERE** | | **GRADE 4**  **Potentially Life Threatening** |
| **HAEMATOLOGY *Standard International Units are listed in italics*** | | | | | | | | | |
| Absolute CD4+ count  **Adult and Pediatric  > 13 years**  (HIV negative only) | | | 300 – 400/mm³  *300 – 400/µL* | | 200 – 299/mm³  *200 – 299/µL* | | 100 – 199/mm³  *100 – 199/µL* | | < 100/mm³  *< 100/µL* |
| Absolute lymphocyte count  **Adult and Pediatric > 13 years** (HIV negative only) | | | 600 – 650/mm³  *0.600 x 10^9^ – 0.650 x 10^9^/L* | | 500 – 599/mm³  *0.500 x 10^9^ – 0.599 x 10^9^/L* | | 350 – 499/mm³  *0.350 x 10^9^ – 0.499 x 10^9^/L* | | < 350/mm³  *< 0.350 x 10^9^/L* |
| Absolute neutrophil count (ANC) | | | | | | | | | |
|  | **Adult and Pediatric > 7 days** | 1,000 – 1,300/mm³  *1.000 x 10^9^ – 1.300 x 10^9^/L* | | 750 – 999/mm³  *0.750 x 10^9^ – 0.999 x 10^9^/L* | | 500 – 749/mm³  *0.500 x 10^9^ – 0.749 x 10^9^/L* | | < 500/mm³  *< 0.500 x 10^9^/L* | |
|  | **Infant**^a,b^  **2 – ≤ 7 days** | 1,250 – 1,500/mm³  *1.250 x 10^9^ – 1.500 x 10^9^/L* | | 1,000 – 1,249/mm³  *1.000 x 10^9^ –  1.249 x 10^9^/L* | | 750 – 999/mm³  *0.750 x 10^9^ – 0.999 x 10^9^/L* | | < 750/mm³  *< 0.750 x 10^9^/L* | |
|  | **Infant**^a,b^ **1 day** | 4,000 – 5,000/mm³  *4.000 x 10^9^ – 5.000 x 10^9^/L* | | 3,000 – 3,999/mm³  *3.000 x 10^9^ – 3.999 x10^9^/L* | | 1,500 – 2,999/mm³  *1.500 x 10^9^ – 2.999 x 10^9^/L* | | < 1,500/mm³  *< 1.500 x 10^9^/L* | |
| Fibrinogen, decreased | | 100 – 200 mg/dL  *1.00 – 2.00 g/L*  OR  0.75 – 0.99 x LLN | | 75 – 99 mg/dL  *0.75 – 0.99 g/L*  OR  0.50 – 0.74 x LLN | | 50 – 74 mg/dL  *0.50 – 0.74 g/L*  OR  0.25 – 0.49 x LLN | | < 50 mg/dL  *< 0.50 g/L*  OR  < 0.25 x LLN  OR Associated with gross bleeding | |
| Haemoglobin (Hgb)^c^ | | | | | | | | | |
|  | **Adult and Pediatric**  **≥ 57 days** (HIV positive only) | 8.5 – 10.0 g/dL  *5.2 – 6.1 mmol/L* | | 7.5 – 8.4 g/dL  *4.6 – 5.1 mmol/L* | | 6.50 – 7.4 g/dL  *3.9 – 4.5 mmol/L* | | < 6.5 g/dL  *< 3.9 mmol/L* | |
|  | **Adult and Pediatric**  **≥ 57 days**  (HIV negative only) | 10.0 – 10.9 g/dL  *6.1 – 6.6 mmol/L*  OR  Any decrease  2.5 – 3.4 g/dL  *1.5 – 2.0 mmol/L* | | 9.0 – 9.9 g/dL  *5.5 – 6.0 mmol/L*  OR  Any decrease  3.5 – 4.4 g/dL  *2.1 – 2.6 mmol/L* | | 7.0 – 8.9 g/dL  *4.2 – 5.4 mmol/L*  OR  Any decrease  ≥ 4.5 g/dL  *≥ 2.7 mmol/L* | | < 7.0 g/dL  *< 4.2 mmol/L* | |
|  | **Infant**^a,b^ **36 – 56 days**  (HIV positive or negative) | 8.5 – 9.4 g/dL  *5.2 – 5.7 mmol/L* | | 7.0 – 8.4 g/dL  *4.1 – 5.1 mmol/L* | | 6.0 – 6.9 g/dL  *3.6 – 4.2 mmol/L* | | < 6.00 g/dL  *< 3.6 mmol/L* | |

^a^ Values are for term infants.

^b^  Use age and sex appropriate values (e.g., bilirubin), including preterm infants.

^c^ Revised by Tibotec Pharmaceuticals Ltd.; monomer conversion factor used for conversion from g/dL to mmol/L

| **LABORATORY** | | | | | | |
| --- | --- | --- | --- | --- | --- | --- |
| **PARAMETER** | | | **GRADE 1**  **MILD** | **GRADE 2**  **MODERATE** | **GRADE 3**  **SEVERE** | **GRADE 4**  **POTENTIALLY LIFE-THREATENING** |
|  | **Infant**^a, b^ **22 – 35 days**  (HIV positive or negative) | | 9.5 – 10.5 g/dL  *1.47 – 1.63 mmol/L* | 8.0 – 9.4 g/dL  *1.24 – 1.46 mmol/L* | 7.0 – 7.9 g/dL  *1.09 – 1.23 mmol/L* | < 7.00 g/dL  *< 1.09 mmol/L* |
|  | **Infant**^a, b^ **1 – 21 days**  (HIV positive or negative) | | 12.0 – 13.0 g/dL  *1.86 – 2.02 mmol/L* | 10.0 – 11.9 g/dL  *1.55 – 1.85 mmol/L* | 9.0 – 9.9 g/dL  *1.40 – 1.54 mmol/L* | < 9.0 g/dL  *< 1.40 mmol/L* |
| International normalized ratio of prothrombin time  (INR) | | | 1.1 – 1.5 x ULN | 1.6 – 2.0 x ULN | 2.1 – 3.0 x ULN | > 3.0 x ULN |
| Methaemoglobin | | | 5.0% – 10.0% | 10.1% – 15.0% | 15.1% – 20.0% | > 20.0% |
| Prothrombin time (PT) | | | 1.1 – 1.25 x ULN | 1.26 – 1.50 x ULN | 1.51 – 3.00 x ULN | > 3.00 x ULN |
| Partial thromboplastin  time (PTT) | | | 1.1 – 1.66 x ULN | 1.67 – 2.33 x ULN | 2.34 – 3.00 x ULN | > 3.00 x ULN |
| Platelets, decreased | | | 100,000 – 124,999/mm³  *100.000 x 10^9^ – 124.999 x 10^9^/L* | 50,000 – 99,999/mm³  *50.000 x 10^9^– 99.999 x 10^9^/L* | 25,000 – 49,999/mm³  *25.000 x 10^9^– 49.999 x 10^9^/L* | < 25,000/mm³  *< 25.000 x 10^9^/L* |
| WBC, decreased | | | 2,000 – 2,500/mm³  *2.000 x 10^9^ – 2.500 x 10^9^/L* | 1,500 – 1,999/mm³  *1.500 x 10^9^– 1.999 x 10^9^/L* | 1,000 – 1,499/mm³  *1.000 x 10^9^– 1.499 x 10^9^/L* | < 1,000/mm³  *< 1.000 x 10^9^/L* |
| **CHEMISTRIES  *Standard International Units are listed in italics*** | | | | | | |
| Acidosis | | | NA | pH < normal, but ≥ 7.3 | pH < 7.3 without life-threatening  consequences | pH < 7.3 with life-threatening  consequences |
| Albumin, serum, low | | | 3.0 g/dL – < LLN  *30 g/L* – *< LLN* | 2.0 – 2.9 g/dL  *20 – 29 g/L* | < 2.0 g/dL  *< 20 g/L* | NA |
| Alkaline phosphatase | | | 1.25 – 2.5 x ULN^b^ 156 – 324 U/L^b^ | 2.6 – 5.0 x ULN^b^ 325 – 625 U/L^b^ | 5.1 – 10.0 x ULN^b^ 626 – 1250 U/L^b^ | > 10.0 x ULN^b^ > 1250 U/L^b^ |
| Alkalosis | | | NA | pH > normal, but ≤ 7.5 | pH > 7.5 without lifethreatening  consequences | pH > 7.5 with lifethreatening  consequences |
| ALT (SGPT) | | | 1.25 – 2.5 x ULN | 2.6 – 5.0 x ULN | 5.1 – 10.0 x ULN | > 10.0 x ULN |
| AST (SGOT) | | | 1.25 – 2.5 x ULN | 2.6 – 5.0 x ULN | 5.1 – 10.0 x ULN | > 10.0 x ULN |
| Bicarbonate, serum, low | | | 16.0 mEq/L – < LLN  *16.0 mmol/L – < LLN* | 11.0 – 15.9 mEq/L  *11.0 – 15.9 mmol/L* | 8.0 – 10.9 mEq/L  *8.0 – 10.9 mmol/L* | < 8.0 mEq/L  *< 8.0 mmol/L* |
| Bilirubin (Total) | | | | | | |
|  | | **Adult and Pediatric  >14 days** | 1.1 – 1.5 x ULN | 1.6 – 2.5 x ULN | 2.6 – 5.0 x ULN | > 5.0 x ULN |

^a^ Values are for term infants.
^b^  Use age and sex appropriate values (e.g., bilirubin), including preterm infants.

| **LABORATORY** | | | | | |
| --- | --- | --- | --- | --- | --- |
| **PARAMETER** | | **GRADE 1**  **MILD** | **GRADE 2**  **MODERATE** | **GRADE 3**  **SEVERE** | **GRADE 4**  **POTENTIALLY LIFE-THREATENING** |
|  | **Infant**^a, b^ **≤ 14 days** (non-haemolytic) | NA | 20.0 – 25.0 mg/dL  *342 – 428 µmol/L* | 25.1 – 30.0 mg/dL  *429 – 513 µmol/L* | > 30.0 mg/dL  *> 513.0 µmol/L* |
|  | **Infant**^a, b^ **≤ 14 days** (haemolytic) | NA | NA | 20.0 – 25.0 mg/dL  *342 – 428 µmol/L* | > 25.0 mg/dL  *> 428 µmol/L* |
| Calcium, serum, high (corrected for albumin) | | | | | |
|  | **Adult and Pediatric ≥ 7 days** | 10.6 – 11.5 mg/dL  *2.65 – 2.88 mmol/L* | 11.6 – 12.5 mg/dL  *2.89 – 3.13 mmol/L* | 12.6 – 13.5 mg/dL  *3.14 – 3.38 mmol/L* | > 13.5 mg/dL  *> 3.38 mmol/L* |
|  | **Infant**^a, b^ **< 7 days** | 11.5 – 12.4 mg/dL  *2.88 – 3.10 mmol/L* | 12.5 – 12.9 mg/dL  *3.11 – 3.23 mmol/L* | 13.0 – 13.5 mg/dL  *3.245 – 3.38 mmol/L* | > 13.5 mg/dL  *> 3.38 mmol/L* |
| Calcium, serum, low (corrected for albumin) | | | | | |
|  | **Adult and Pediatric ≥ 7 days** | 7.8 – 8.4 mg/dL  *1.95 – 2.10 mmol/L* | 7.0 – 7.7 mg/dL  *1.75 – 1.94 mmol/L* | 6.1 – 6.9 mg/dL  *1.53 – 1.74 mmol/L* | < 6.1 mg/dL  *< 1.53 mmol/L* |
|  | **Infant**^a, b^ **< 7 days** | 6.5 – 7.5 mg/dL  *1.63 – 1.88 mmol/L* | 6.0 – 6.4 mg/dL  *1.50 – 1.62 mmol/L* | 5.50 – 5.90 mg/dL  *1.38 – 1.51 mmol/L* | < 5.50 mg/dL  *< 1.38 mmol/L* |
| Cardiac troponin I (cTnI) | | NA | NA | NA | Levels consistent with myocardial infarction or unstable angina as defined by the manufacturer |
| Cardiac troponin T (cTnT) | | NA | NA | NA | ≥ 0.20 ng/mL OR  Levels consistent with myocardial infarction or unstable angina as defined by the manufacturer |
| Cholesterol (fasting) | | | | | |
|  | **Adult ≥ 18 years** | 200 – 239 mg/dL  *5.18 – 6.19 mmol/L* | 240 – 300 mg/dL  *6.20 – 7.77 mmol/L* | > 300 mg/dL  *> 7.77 mmol/L* | NA |
|  | **Pediatric  < 18 years** | 170 – 199 mg/dL  *4.40 – 5.15 mmol/L* | 200 – 300 mg/dL  *5.16 – 7.77 mmol/L* | > 300 mg/dL  *> 7.77 mmol/L* | NA |
| Creatine kinase | | 3.0 – 5.9 x ULN^b^ | 6.0 – 9.9 x ULN^b^ | 10.0 – 19.9 x ULN^b^ | **≥** 20.0 x ULN^b^ |
| Creatinine | | 1.1 – 1.3 x ULN^b^ | 1.4 – 1.8 x ULN^b^ | 1.9 – 3.4 x ULN^b^ | **≥** 3.5 x ULN^b^ |
| Glucose, serum, high | | | | | |
|  | Nonfasting | 116 – 160 mg/dL  *6.44* – *8.88 mmol/L* | 161 – 250 mg/dL  *8.89 – 13.88 mmol/L* | 251 – 500 mg/dL  *13.89 – 27.75 mmol/L* | > 500 mg/dL  *> 27.75 mmol/L* |
|  | Fasting | 110 – 125 mg/dL  *6.11 – 6.94 mmol/L* | 126 – 250 mg/dL  *6.95 – 13.88 mmol/L* | 251 – 500 mg/dL  *13.89 – 27.75 mmol/L* | > 500 mg/dL  *> 27.75 mmol/L* |
| Glucose, serum, low | | | | | |
|  | **Adult and Pediatric  ≥ 1 month** | 55 – 64 mg/dL  *3.05 – 3.55 mmol/L* | 40 – 54 mg/dL  *2.22 – 3.06 mmol/L* | 30 – 39 mg/dL  *1.67 – 2.23 mmol/L* | < 30 mg/dL  *< 1.67 mmol/L* |
|  | **Infant**^a,b^ **< 1 month** | 50 – 54 mg/dL  *2.78 – 3.00 mmol/L* | 40 – 49 mg/dL  *2.22 – 2.77 mmol/L* | 30 – 39 mg/dL  *1.67 – 2.21 mmol/L* | < 30 mg/dL  *< 1.67 mmol/L* |

^a^ Values are for term infants.
^b^  Use age and sex appropriate values (e.g., bilirubin), including preterm infants.

| **LABORATORY** | | | | | | |
| --- | --- | --- | --- | --- | --- | --- |
| **PARAMETER** | | **GRADE 1**  **MILD** | **GRADE 2**  **MODERATE** | | **GRADE 3**  **SEVERE** | **GRADE 4**  **POTENTIALLY LIFE-THREATENING** |
| Lactate | | < 2.0 x ULN without acidosis | ≥ 2.0 x ULN without acidosis | | Increased lactate with pH < 7.3 without life-threatening consequences | Increased lactate with pH < 7.3 with life-threatening consequences |
| LDL cholesterol (fasting) | | | | | | |
|  | **Adult ≥ 18 years** | 130 – 159 mg/dL  *3.37* **–** *4.12 mmol/L* | 160 – 190 mg/dL  *4.13 – 4.90 mmol/L* | | ≥ 190 mg/dL  *≥ 4.91 mmol/L* | NA |
|  | **Pediatric  > 2** *–* **< 18 Years** | 110 – 129 mg/dL  *2.85 – 3.34 mmol/L* | 130 – 189 mg/dL  *3.35 – 4.90 mmol/L* | | ≥ 190 mg/dL  *≥ 4.91 mmol/L* | NA |
| Lipase | | 1.1 – 1.5 x ULN | 1.6 – 3.0 x ULN | | 3.1 – 5.0 x ULN | > 5.0 x ULN |
| Magnesium, serum, low | | 1.2 – 1.4 mEq/L  *0.60 – 0.70 mmol/L* | 0.9 – 1.1 mEq/L  *0.45 – 0.59 mmol/L* | | 0.6 – 0.8 mEq/L  *0.30 – 0.44 mmol/L* | < 0.60 mEq/L  *< 0.30 mmol/L* |
| Pancreatic amylase | | 1.1 – 1.5 x ULN | 1.6 – 2.0 x ULN | | 2.1 – 5.0 x ULN | > 5.0 x ULN |
| Phosphate, serum, low | | | | | | |
|  | **Adult and Pediatric > 14 years** | 2.5 mg/dL – < LLN  *0.81 mmol/L* – *< LLN* | 2.0 – 2.4 mg/dL  *0.65 – 0.80 mmol/L* | | 1.0 – 1.9 mg/dL  *0.32 – 0.64 mmol/L* | < 1.00 mg/dL  *< 0.32 mmol/L* |
|  | **Pediatric  1 – 14 years** | 3.0 – 3.5 mg/dL  *0.97 – 1.13 mmol/L* | 2.5 – 2.9 mg/dL  *0.81 – 0.96 mmol/L* | | 1.5 – 2.4 mg/dL  *0.48 – 0.80 mmol/L* | < 1.50 mg/dL  *< 0.48 mmol/L* |
|  | **Pediatric < 1 year** | 3.5 – 4.5 mg/dL  *1.13 – 1.45 mmol/L* | 2.5 – 3.4 mg/dL  *0.81 – 1.12 mmol/L* | | 1.5 – 2.4 mg/dL  *0.48 – 0.80 mmol/L* | < 1.50 mg/dL  *< 0.48 mmol/L* |
| Potassium, serum, high | | 5.6 – 6.0 mEq/L  *5.6* – *6.0 mmol/L* | 6.1 – 6.5 mEq/L  *6.1 – 6.5 mmol/L* | | 6.6 – 7.0 mEq/L  *6.6 – 7.0 mmol/L* | > 7.0 mEq/L  *> 7.0 mmol/L* |
| Potassium, serum, low | | 3.0 – 3.4 mEq/L  *3.0 – 3.4 mmol/L* | 2.5 – 2.9 mEq/L  *2.5 – 2.9 mmol/L* | | 2.0 – 2.4 mEq/L  *2.0 – 2.4 mmol/L* | < 2.0 mEq/L  *< 2.0 mmol/L* |
| Sodium, serum, high | | 146 – 150 mEq/L  *146 – 150 mmol/L* | 151 – 154 mEq/L  *151 – 154 mmol/L* | | 155 – 159 mEq/L  *155 – 159 mmol/L* | ≥ 160 mEq/L  *≥ 160 mmol/L* |
| Sodium, serum, low | | 130 – 135 mEq/L  *130 – 135 mmol/L* | 125 – 129 mEq/L  *125 – 129 mmol/L* | | 121 – 124 mEq/L  *121 – 124 mmol/L* | ≤ 120 mEq/L  *≤ 120 mmol/L* |
| Triglycerides (fasting) | | NA | 500 – 750 mg/dL  *5.65 – 8.48 mmol/L* | | 751 – 1,200 mg/dL  *8.49 – 13.56 mmol/L* | > 1,200 mg/dL  *> 13.56 mmol/L* |
| Uric acid | | 7.5 – 10.0 mg/dL  *0.45 – 0.59 mmol/L* | 10.1 – 12.0 mg/dL  *0.60 – 0.71 mmol/L* | | 12.1 – 15.0 mg/dL  *0.72 – 0.89 mmol/L* | > 15.0 mg/dL  *> 0.89 mmol/L* |
| **URINALYSIS  *Standard International Units are listed in italics*** | | | | | | |
| Haematuria (microscopic) | | 6 – 10 RBC/HPF | > 10 RBC/HPF | Gross, with or without clots OR with RBC casts | | Transfusion indicated |
| Proteinuria, random collection | | 1 + | 2 – 3 + | 4 + | | NA |
| Proteinuria, 24 hour collection | | | | | | |
|  | **Adult and Pediatric  ≥** **10 years** | 200 – 999 mg/24 h  *0.200 – 0.999 g/d* | 1,000 – 1,999 mg/24 h  *1.000 – 1.999 g/d* | 2,000 – 3,500 mg/24 h  *2.000 – 3.500 g/d* | | > 3,500 mg/24 h  *> 3.500 g/d* |
|  | **Pediatric  > 3 months** *–*  **< 10 years** | 201 – 499 mg/m2/24 h  *0.201 – 0.499 g/d* | 500 – 799 mg/m2/24 h  *0.500 – 0.799 g/d* | 800 – 1,000 mg/m2/24 h  *0.800 – 1.000 g/d* | | > 1,000 mg/ m2/24 h  *> 1.000 sg/d* |

^a^ Values are for term infants.
^b^  Use age and sex appropriate values (e.g., bilirubin), including preterm infants.

**Appendix III: Schedule of Events**

| Evaluation | Screening  (within 60 days) |  |  | | |  | |  | | On-Study Evaluations (days) | | | | | | | | | | | Premature Treatment Disc. |
| --- | --- | --- | --- | --- | --- | --- | --- | --- | --- | --- | --- | --- | --- | --- | --- | --- | --- | --- | --- | --- | --- |
|  |  | Baseline Day 0 | | 1 | 2-3 | | 4 – 5 * | | 6 | | 7 | 8-10 | 11 – 12* | 13 | 14 | 15-16 | 17-28* | 29 | 30 | Follow-up (+7 days) |  |
| Informed Consent | X |  | |  |  | |  | |  | |  |  |  |  |  |  |  |  |  |  |  |
| HIV, Hepatitis B and C | X |  | |  |  | |  | |  | |  |  |  |  |  |  |  |  |  |  |  |
| Medical History | X |  | |  |  | |  | |  | |  |  |  |  |  |  |  |  |  |  |  |
| Medication History | X |  | |  |  | |  | |  | |  |  |  |  |  |  |  |  |  |  |  |
| Complete Physical Exam | X |  | |  |  | |  | |  | |  |  |  |  |  |  |  |  |  |  |  |
| Concomitant Medications |  | X | |  |  | |  | | X | |  |  |  | X |  |  |  | X |  |  |  |
| Targeted Physical Exam | X |  | | X |  | |  | |  | | X |  |  |  | X |  |  |  | X | X | X |
| Vital signs | X |  | | X |  | |  | |  | | X |  |  |  | X |  |  |  | X | X | X |
| Height/Weight | X |  | |  |  | |  | |  | |  |  |  |  |  |  |  |  |  |  |  |
| Haematology | X |  | | X |  | |  | |  | | X |  |  |  | X |  |  |  | X | X | X |
| Chemistry | X |  | | X |  | |  | |  | | X |  |  |  | X |  |  |  | X | X | X |
| Genotyping | X |  | |  |  | |  | |  | |  |  |  |  |  |  |  |  |  |  |  |
| CK, Lipase, Pancreatic Amylase | X |  | |  |  | |  | |  | |  |  |  |  |  |  |  |  |  |  |  |
| Urinalysis | X |  | |  |  | |  | |  | |  |  |  |  |  |  |  |  |  |  |  |
| Urine B-HCG (WOCBP) | X |  | |  |  | |  | |  | |  |  |  |  |  |  |  |  |  |  |  |
| Inhibitor (RTV 100 mg BD or KCZ 200 mg BD) Dosing |  |  | |  |  | | X1 | | X1 | | X1 |  | X2 | X2 | X2 |  |  |  |  |  |  |
| Inducer Dosing (RIF 600 mg ON) |  |  | |  |  | |  | |  | |  |  |  |  |  |  | X | X | X |  |  |
| Pharmacokinetic Sampling (MDZ and RAL) |  |  | | X |  | |  | |  | | X |  |  |  | X |  |  |  | X |  |  |
| Review of Medication Diary |  |  | |  |  | | X | | X | |  |  |  | X |  |  |  | X |  |  | X |
| Pill Counts |  |  | |  |  | |  | | X | |  |  |  | X |  |  |  | X |  |  | X |
| Adverse Event Assessment |  |  | |  |  | | X | | X | | X |  | X | X | X |  | X | X | X | X | X |

* - Allowed up to 3 days later
